# Analectic

**Published:** 1832

**Authors:** 


### ANALECTIC, ANALYTICAL AND ORIGINAL.

### I. ANALECTIC.

1. *Process of preparing pure Muriate of Morphia.* By Montgomery Robertson.—"Having now finished my illustrations of its Medicinal effects in a state of purity, I shall direct the attention of my readers to what appears to me the best mode of securing this, and the readiest method of ascertaining it. And as I intend subsequently, to point out a way of obtaining, not the muriate only, but any other salt of Morphia, in a state of purity, and in as far as its individual composition will allow, of uniformity also; I shall take a brief view of former processes, that the value of the improvements may be more clearly seen.

"Until the introduction of the muriate into medicine, the salts of morphia were prepared on a plan divisible into four stages; which had for their ostensible objects, the separation of the active from the inert principles,—the isolation of the morphia,—its decolorization,—and its combination with acids. The first was accomplished by dissolving the salts contained in opium,—the next by decomposing them with an alkali,—the third by treating the precipitate with charcoal and alcohol, and the last by dissolving the product in an acid. Latterly, comparative purity had been obtained by decolorizing a salt of morphia, which was afterwards decomposed to procure the Alkaloid for recombination. The first step has been effected at different periods by means of water, hot or cold, acidulated or plain; or lastly, by fermentation. In whatever way it is made, the infusion contains a free acid, more or less resinous coloring matter, solutions of morphia and of narcotine. The three last, intimately blended, are thrown down on the addition of ammonia; animal charcoal, and repeated crystallizations in alcohol, remove the coloring resin; but the narcotine passes untouched through all these ordeals, and from its similarity to morphia, has been generally mistaken for it. This impure product being dissolved in the acetic or sulphuric acids, the liquid is evaporated to dryness; and thus the narcotine remains mixed with the resulting salt, either in a crystalline form, (if, as Robiquet asserts, it be crystallizable,) or in the shape of a transparent coating, enveloping its particles.

"Some chemists, indeed, as Merk and Wittstock, have, by ingenious and expensive processes, obtained morphia perfectly pure. But I speak of the alkaloid as it is generally manufactured; when the object of the pharmaceutist being merely to procure a saleable article, purity is little valued, and not particularly sought after, when it is obtained at the expense of considerable diminution in quantity.

"It is easy to observe that the complication of treatment which these processes require; the combinations, decompositions, solutions, and crystallizations which they enjoin; the evaporations and filtrations which they direct, cannot be completed without considerable expense of time, labor, and material. But by introducing into medicine the muriate of morphia, and publishing a mode of preparing it, Dr. Gregory rendered the latter part of these processes useless, and abridged much that was tedious and objectionable. I have now to propose an improved method; which, by curtailing still further the necessary operations, will render the preparation of the muriate, much more easy and economical.

"This process has for its prominent feature the employment of *double* decomposition; a plan which secures at one step the separation of the meconic acid,—and the union of the morphia with muriatic acid. The liquid employed to effect this decomposition is the muriate of lime; an article whose cheapness renders economy in its use no object. Perhaps in other places, muriates may be found more suitable, for other reasons, but this one seems to me best calculated to enable us to procure, on the one hand the meconic acid free from color, on the other the muriate of morphia free from narcotine.

"The first step of the process resembles that of all others, in as far as the active principles of the solution is concerned. The opium, cut in pieces, is macerated at a temperature not exceeding 100 deg. F.; as it softens, it is worked up into a pulp, and frequently stirred during the course of exhaustion; the infusion, as it becomes saturated is drawn off clear, and may be immediately subjected to evaporation.

"This evaporation is conducted in a large vessel of tinned iron; and a sufficient quantity of marble, in coarse powder, is added to saturate the free acid. The muriate of lime used should contain no iron, lest, in combination with meconic acid, it give a deep color to the liquid, difficult to be got rid off, and the quantity required for each portion of infusion, is best learned by experience. When the infusion has reached the consistence of syrup, an excess of muriate of lime is poured in, and the boiling is continued for a few minutes longer. Then the whole may be emptied into a large basin, and when cold diluted with water, until a copious separation of resinous flocculi take place. In this way most of the meconate of lime, which is nearly insoluble, and a great quantity of coloring matter, are got rid of; the separation of the latter being more copious and complete, the more concentrated the liquid is before dilution, and the nearer that dilution approaches to a certain point; after which, further dilution causes the flocculi to be partially redissolved, and to render the filtered liquid turbid.

"When these flocculi have subsided, the clear part is evaporated as far as possible, on a sand-bath, a small bit of marble being put into each dish to neutralize any free acid, and the fluid being poured off the sediment before it is permitted to crystallize. We may, at this stage, prove whether enough muriate of lime has been added, by trying if a little of the clear liquid will, aided by heat, separate meconate of lime from an equal bulk of concentrated infusion. When about to consolidate, it is stirred into a uniform mass, from which, enclosed in a stout cloth, the dark fluid is expressed as completely as possible. The cake of impure muriate is dissolved in water at 70 deg. F., and the solution filtered through cloth; whereby a large quantity of impurities will be separated without loss; the liquid, to which a small portion of muriate of lime is added, is now ree-vaporated, neutralized, and treated in other respects as before. In the next evaporation the liquid, now completely free from meconate of lime is slightly acidulated; a judicious suggestion due to Dr. Gregory, who has observed that the acid renders the coloring matter more soluble, aud thus more completely separated, when the product is expressed for the third time. The muriate, which is now of a light brown color, is next dissolved in boiling water carefully neutralized with chalk, and mixed with animal charcoal; which, provided it do not contain a free alkali, requires no purification. Fresh portions of hot water should, from time to time, be added, until there is enough to keep the salt in solution, when the liquid cools; and the whole is to be frequently stirred, that the most may be made of every particle of charcoal. The temperature should not permanently exceed 190 deg. F. lest any of the muriate be decomposed. If the charcoal be good, and in sufficient quantity at the same time that the liquid is dilute, and frequently agitated, twenty-four hours of contact is sufficient to decolorize it so far, that on the addition of a little acid to the filtered liquid, it becomes almost colorless. This effect produced on the color by the addition of an acid, (any acid will do,) I cannot explain. It was first observed by Dr. Gregory, who has also remarked that muriatic acid added to a neutral solution of muriate of the specific gravity of 1.020, when cold, and in which there is no appearance of crystallization, causes it in a short time to become a mass of crystals, which, when dried in the air, are perfectly neutral.

"The mass that results from the evaporation of the decolorized liquid, is subjected to pressure in portions of about six ounces each, tied up in well washed cotton cloth; the superfluous ends of which are cut off, in order to prevent them from making the surface of the cake irregular. They are next put into a stove heated to about 100 deg. F. where they remain until almost dry, when the cloth is removed and the colored surface scraped off. After being pounded and dried, *till no more weight is lost* at a temperature of 150 deg. F. The muriate resembles chalk in color and appearance, is permanent in the air, and is fit for medical use. If a little be now dissolved in distilled water, and pure potash added, crystals of morphia are presently separated, which are completely dissolved on the addition of an excess of the alkali, the absence of the characteristic milkiness, changed by heat to woolly flocks, indicating a total freedom from narcotine. A very few colored flocculi may remain, so inconsiderable in weight as to be of no moment, and totally removed by filtration; as in any remaining yellow tinge by a fresh crystallization, if the liquid be previously well acidulated.

"In this process, if well conducted, the loss by manipulation is trifling. The whole of the morphia is separated in a solid form in the first and second crystallizations, provided there be present in the liquid a slight excess of muriate of lime, and the evaporation be carried far enough. The great deliquescence of the muriate of lime apparently facilitates the completeness of this separation. The dark liquids expressed on these two occasions may therefore be deemed void of muriate; the mother liquids of future crystallizations, as well as the water in which the cloths have been rinsed, are added to a portion in an earlier stage of advancement, and the colored particles scraped from the cakes, may be joined to that about to be treated with charcoal, it is absolutely necessary that all the neutralizations be made without lime, (marble will not decompose the warm liquid, nor chalk the cold,) —that all the evaporations be carried as far as possible before setting aside to crystallize—that the mass be always stirred when consolidating—and that the mother liquids be expressed very completely from the crystals, enclosed in cloth. The charcoal does no good until most of the resin be separated, and leaves a dark tinge in the liquid, only to be removed by acidulating. A moderate excess of muriate of lime is proper, too much renders the mass clammy, and the mother liquid difficult to express; too little causes the decomposition to be incomplete, and the muriate that is formed refuses to crystallize; but these errors are easily remedied.

"In the last experiment that I made, 54 1-2 lbs. Avoird. of good opium were macerated at once. This quantity had lost one pound and a half of its weight previous to infusion, by standing for three months in the laboratory, having been previously reckoned 56 lbs. From it were obtained, by the process just described, 91 1-2 ounces troy of muriate, or rather more than 11 1-2 per cent. Several accidents happened during the preparation, from the breaking of dishes; besides the opium was not completely exhausted, nor were the mother liquids collected with due regard to economy, or I have no doubt the other half per cent. might easily have been made out; and 12 per cent. is in my opinion the utmost that can be expected from ordinary opium, losing from 8 to 9 per cent. by drying. The cost of manufacture, including the price of the opium, &c. amounted to 17s. 6d. the ounce troy. This then may be reckoned a fair trial of its success on a large scale.

"With regard to the adulteration of this article, for I fear that adulteration, though easy of detection, will still be attempted; the most likely manner of increasing its weight will be, not to deprive it completely of moisture. This may be detected, simply by ascertaining how far heat lessens the weight of a certain number of grains, and druggists are entreated to take particular care to avoid using muriate that is not perfectly dry; as this, which may proceed from accident as well as design, is, more than any other cause, likely to impair the uniformity of its preparations. In the state of powder it may, perhaps, be mixed with chalk, magnesia, starch, carbonate of baryta, sugar, and other white powders; a species of adulteration which may be completely prevented by preparing it in crystals. But as this requires more time and manipulation, I may here observe, that all insoluble powders are detected by its solubility in hot water, starch by iodine, and sugar by the taste, and the form of its crystals on evaporation. I may here too mention, that I have seen in chemical works, particular cautions given to avoid the fumes of boiling infusions of opium, as if they were highly deleterious. But though engaged for several months in examining the component ingredients of opium, and though I have been exposed for hours, together with the concentrated vapors of infusions, I have never experienced the slightest inconvenience therefrom; nor do I believe there is the least particle of narcotic principle contained in them.

"These are all the particulars I have to mention with regard to the manufacture of the muriate of morphia. I come now to describe a method, by which any other of its salts may be prepared in a state of purity, from crystals of impure morphia.

"For the principle on which this process is founded, I am indebted to M. Buisson of Paris, who states in his Thesis, that the salts of ammonia are decomposed at an elevated temperature, by the salifiable vegetable bases. On trial, I found that morphia, boiled in a salt of ammonia, displaced the volatile alkali, while narcotine had no such power; and that when morphia, mixed with narcotine, was boiled in muriate of ammonia, until no more ammonia was driven off, the narcotine was left undissolved in the form of powder, while the morphia had disappeared. In order then to form a pure salt of morphia from a mixture of the alkaloids, I boil them in a little distilled water, dropping in, carefully, a neutral solution of the acid in combination with ammonia, until, on a further addition, no more fumes of ammonia are exhaled. The liquid is then filtered to separate the narcotine; the morphia may be obtained by precipitation. It is proper to remark, that the crystals must be tolerably free from resin, in order that a perfect separation take place; and that the coloring matter, also, seems to have the power of decomposing the ammoniacal salt. This process may be hereafter useful in rendering narcotine pure, a principle, which, it is probable, will, ere long become more esteemed in medicine, than it has hitherto been. It may be modified in various ways to suit the convenience of the operator, and is a great aid in the examination of small portions of opium; as in estimating the value of a sample.—*Ed. Med. and Surg. Journ. April*, 1832.

2. *Poisoning by Colchicum.* By Thomas Ferredy.—David Cole, æt. 44, a stout, muscular man, feeling pains in his bowels, to which he was subject, about six o'clock in the morning of the 8th of March last, swallowed, believing it to be rum, about two oz. of wine of the seeds of colchicum. He immediately discovered his error, but, knowing its effects in a small dose, conceived it would be followed by vomiting and purging sufficient to avert mischief. He sought no medical advice until four in the afternoon, when I first saw him. He was sitting in a chair, his elbows on his knees, and told me that he felt no inconvenience for an hour and a half after taking the dose, when pains in the bowels came on, but that he continued his labor until eleven o'clock, when pains in his stomach and bowels, retching, and copious vomiting of a yellowish fluid compelled him to desist.

*Four o'clock, P. M.*—He describes the pain in the epigastrium as agonizing, and says it is like a knife piercing him. The retching is incessant and extremely violent, but no fluid is evacuated; there is tenesmus; a small quantity of fæcal matter only has passed. No tenderness upon pressure either in the epigastrium or abdomen. The appearance of the tongue is natural, the pulse small, slow, and feeble; breathing not much affected; the feet cold; his countenance is anxious; features sharp; his cheeks, lips, and palpebræ, purple. Upon attempting to walk, says he thinks he shall lose the use of his limbs. A mustard emetic was given, followed by copious draughts of warm water and gruel. These were soon returned, with apparently no mixture. Cathartic medicine was given, and immediately returned. Was put to bed; warm bricks were applied to the feet, and hot flannels to the stomach. To take forty drops of laudanum immediately; gruel and coffee plenteously.

*Nine o'clock, P. M.*—The retching, vomiting, and pain in the stomach, continue with undiminished violence; the fluid vomited contains a sediment like coffee-grounds; very thirsty, has made little water. Twenty drops of laudanum every two hours; a blister to the epigastrium; sinapisms to the feet; an enema every hour.

*9th—Six o'clock, A. M.*—Has passed a sleepless night; the symptoms remain unaltered. The eyes are sunk; the feet warmer; skin generally natural; no perspiration; pulse scarcely to be felt; respiration hurried; great thirst; no urine. Enemata returned without fæcal matter. Camphor, calomel, and opium every three hours; an effervescing draught, with brandy, every hour.

*Eight o'clock, P. M.*—The retchings and pains continued until four o'clock, when the bowels were much distended. Has since had copious liquid stools, dark colored, and very offensive, and expresses himself better. Makes a few drops only with urine; loses his sight for a minute or two after getting out of bed to the night-chair. The pulse is scarcely perceptible and occasionally intermits. He is perfectly sensible, but talks with effort; calls continually for water.—Aromatic confection, carbonate of ammonia and camphor mixture, with brandy, every hour.

10*th.*—In the course of the night his stools passed involuntarily, and in great numbers; his weakness increased, and he died a few minutes before five oclock this morning, perfectly sensible to the last moment.

*Sectio Cadaveris.*—The face, neck, upper and front part of the thorax, insides of the arms, front of each fore-arm, and the insides of the thighs were covered with patches of a purple efflorescence, as were also the integuments of the scrotum and penis. The muscles of the fore-arm were very rigid, and their fibres contracted into hard knobs. The great omentum, instead of covering the front of the intestines, was turned up between the stomach and convex surface of the liver behind, and the diaphragm in front, from the effects of vomiting. There was increased redness in a portion of the peritoneum covering the jejunum. The stomach and bowels were covered with a thick, tenacious, but colorless mucus. On a portion of the mucous membrane of the stomach, near the cardiac orifice, and corresponding to its great arch, was a patch of redness about the size of a half-crown piece; its secretion here did not vary in tenacity, quantity, or color, from that of any other portion of the membrane. Upon dividing it at this part, its section presented nothing beyond its usual appearance; there was no pulpiness, no thickening, but a small quantity of blood was effused between it and the muscular coat, giving a reddened internal appearance. Careful examination of that portion of the reddened peritoneum covering the jejunum demonstrated the like hæmorrhagic condition of the blood-vessels. Blood was effused between the peritoneal and muscular coats, but the mucous membrane corresponding to this portion was perfectly healthy—at least it was perfectly free from inflammation. No other trace of inflammation was observed in any other portion of the abdominal viscera. The gall-bladder was distended with healthy bile: the urinary bladder was contracted and empty.

The pleuræ costales were much reddened. The lungs were of a most beautiful purple color externally, did not crepitate, and were gorged with black blood, which had become effused, underneath the pleuræ pulmonales, in spots of various sizes; these were very numerous about their roots and edges.

The pericardium contained no fluid, nor was it reddened, yet numbers of ecchymosed spots were observed in that portion of it attached to the central tendon of the diaphragm, and also thickly interspersed on the surface of the heart itself, more especially about the centre of the coronary vessels. Heart flabby and easily broken down. The cavæ, the right oracle and ventricle, and the pulmonary artery, were filled with black blood, partly coagulated, partly fluid; the left auricle and ventricle empty.

I regret that neither my own entreaties nor the influence of the coroner were sufficient to obtain permission to examine the head. So little were the functions of the brain disturbed during life, that it is probable no other diseased appearance would have been observed, save a participation of its sinuses in the distension of the right side of the heart.

The deleterious effects of the bulb of colchicum are said to depend, in a great measure, upon the season of the year when it is collected; but the seeds matured only in the spring, must possess uniform properties.

Colchicum is classed, by most writers, as an acrid or rubefacient poison, and poisons of this class are defined as producing inflammation when applied to the intestinal canal, and, if taken in sufficient quantity, the same effects as the corrosive. No such effects however, followed its exhibition in this case; for, in addition to the want of the usual postmortem appearance of inflammation, I consider the patches of redness in the stomach and peritoneum, too partial for inflammation from such a cause. The jejunum, too, possesses a singular immunity from disease as is shown by the quotation of Andral's table, in Macintosh's Practice of Physic. The hæmorrhagic condition of the system, observed here, accords with the observations of Dr. A Thomson, published at p. 281 of the Lancet for 1831, who says, that colchicum has an effect of producing effusion of blood from all the mucous tissues, the skin alone excepted, and that a peculiar laxity is observable in their cellular membrane, producing a diminution, if not total destruction, of its adhesive powers. The purple efflorescence of the skin, the effusions of blood between the coats of the stomach and the jejunum, and underneath the pleuræ and the pericardium, in this case, all prove the accuracy of these remarks. I feel satisfied that not the slightest inflammation existed in either of the above-mentioned structures; for, with respect to the stomach and bowels, where redness was perceptible, the mucous membrane was unchanged in structure; there was neither pulpiness nor thickening, nor did it present a more vascular section than other portions of the membrane: a very important observation, too, its secretion was not changed in quantity or consistence. With respect to the lungs it was difficult to trace, or rather dtstinguish, either blood-vessels or bronchiæ; a section of them seemed more like the section of a clot of venous blood, so greatly were they congested with black blood. The state of the breathing during life bore no kind of proportion to this appearance.

Colchicum is also classed as a poison acting upon the nervous system by absorption, and, through this medium, exerting a peculiar influence on the arterial circulation; and this appears to be its true modus operandi. Sir Everard Home found, by direct experiment, that its effects were the same, whether taken into the stomach or introduced into the jugular vein, only that, in the latter case, its effects were more quickly developed than in the former, and hence he concludes that its action upon the different parts of the body is through the medium of the circulation and not from its immediate effects upon the stomach and intestines. The primæ viæ have been frequently, and perhaps generally, found inflamed, where colchicum has been taken in large quanties; but this is no proof of its *direct* effects upon them, since arsenic will produce inflammation there (the stomach and bowels) when applied to the external surface of the body! Sir Everard Home found that colchicum reduced the pulse in all cases where it was ordinarily used, and this is a material help in enabling us to trace the order in which the three great functions of the body are severally affected by it. It would seem that the heart is deprived of nervous energy, and in consequence beats feebly. Now, by the natural process of inspiration, the trachea and bronchiæ, with their minute ramifications, together with the air-cells, are distended with atmospheric air—an elastic fluid. At the standard of health the heart contracts with a force probably three hundred times greater than the force exerted by the pressure of the air in the lungs; and the blood, instead of being impeded, flows more freely, hence the important connexion between the circulating and respiratory functions. We have seen that colchicum, in an ordinary dose, depresses the heart's action. What must then be expected to follow the administration of a dose like that in question? for it does not appear to be one of those remedies, the principle of whose action is altered by its dose. The action of the heart is almost paralyzed; it can no longer overbalance the pressure exerted by the air in the lungs; the blood can be no longer propelled as usual through their vessels; congestion takes place in the veins; the blood is unarterialized, (and whether its exposure to the action of the fibrillæ of the ganglial system of nerves, in its passage through the arteries, has any influence in effecting this essential change, the lungs are not able to exert their necessary preparatory action,) and unarterialized blood gradually destroys the action of every part through which it circulates. The organs of digestion, assimilation, circulation, and secretion, were severally deranged in function, whilst the intellectual and locomotive powers were little affected. This proves the great extent to which the sympathetic or ganglial system of nerves was affected, and the almost immunity of the cerebro-spinal system.

It must be obvious that no treatment in this case held out any hope of the patient's recovery. When first I saw him, he had swallowed the dose ten hours, when time had been allowed, over and over again, for it to enter the circulation. An emetic, or stomach-pump, employed before this had taken place, would have dislodged it, and probably have saved his life.

Since poisons act in so many different ways upon the animal economy, some primarily through the medium of the nerves, without being absorbed, producing death by suffocation from paralysis of the respiratory muscles, or by syncope—others by entering the circulation, and exerting their influence on the heart, brain, and alimentary canal—others through the same medium on the spinal marrow, and lastly, others having a purely local action on the mucous membrane of the alimentary canal—and since this difference through their physiological actions renders a plan of treatment safe and justifiable in some instances, which would be followed by the most tragic effects in others, it behooves us to be aware of the order of the parts of the system through which they exert their influence; for, as each has its own modus operandi, it is by a knowledge of this only, that we can arrive at scientific and safe indications of cure. Magendie, for instance, found that a state of depletion facilitated absorption, but that a state of congestion impeded it. How important a knowledge of this fact is in the successful treatment of two familiar poisons—arsenic and corrosive sublimate! Dr. Paris has classed the former with those poisons which produce their effects by absorption—the latter with those having a purely local or corrosive action. Both are followed by inflammation of the primæ viæ; yet the experiments of Magendie teach us, that, if we deplete to subdue the inflammatory symptoms when arsenic has been taken, so long as any portion of it remains in the stomach and bowels, we necessarily hasten death by promoting its absorption. On the contrary, corrosive sublimate, having a local action, and not being absorbed, needs no such precaution: we may bleed from the outset.

The same principle is applicable to the poison of colchicum. It produces its effects by absorption. We should, therefore, be very cautious how we bleed, to subdue any inflammatory action of the stomach and bowels, until the poison is completely ejected from them.—*Bost. Med. and Surg. Journ.*

3. *Experiments with Narcotine.* Results of Experiments and Observations on Narcotine.*† By Wm. Tully, M. D. Professor of Materia Medica and Therapeutics in the Medical Institution of Yale College.—It is now many years since the discovery of that proximate principal of Opium commonly called Narcotine, and even a considerable number since it has been known to be capable, in certain doses, of destroying brute animals, with the phenomena usually produced by a *narcotic;* and yet I have no knowledge of any experiments or observations, hitherto, that can be considered as contributing much, if indeed any thing, towards the determination of its medicinal powers upon the human subject. As far as I know, most of the late writers upon Materia Medica have concurred in ascribing all the remedial properties of Opium to some salt of Morphine (another proximate principle of this drug), although it is admitted that Opium contains only about seven *per centum* of Morphine, and that a given quantity of Morphine is very far from being fifteen times as active as the same quantity of Opium, which ought to be the fact were Morphine its sole

* This article is re-published at the author's request, from the American Journal of Science for July. Some future numbers will contain a communication from Dr. Tully, prepared for this Journal, giving the details of these experiments.—*Ed. Bost. Jour.*

† It is well observed by a distinguished pharmaceutist, that the original ending in *ina*, (*ine* in English,) of the names of the vegetable salifiable bases, *must* be retained by physicians to the exclusion of the termination in *a* simply, or *ia*, which has been recently adopted by the chemists, since the similarity of *Atropia* to Atropa—of *Daturia* to Datura—of *Brucia* to Brucca—of *Sanguinara* to Sanguinaria—of *Cinchonia* to Cinchona—of *Gentiania* to Gentiana, etc. (the former of which are the present fashionable names for the active principles, and the latter the long established names for the vegetables from which they are obtained,) is altogether incompatible in prescription with the safety of patients. In all the editions of Magendie's Formulary—in Anthony Todd Thompson's Conspectus of the British Pharmacopœias—in Rennie's Supplement to the British and French Pharmacopœias—and (as has been said) in some of the latest Pharmacopœias of the continent of Europe, the termination in *ina* is retained, it was to be hoped that the responsibility of a useless change in nomenclature which, if adopted, would endanger so many lives, would never be assumed by any practitioner of medicine.

medicinal principle. According to the best observations, a given quantity of Morphine, instead of possessing fifteen times the activity of the same quantity of Opium, can at most be considered as possessing only about four times the activity, and perhaps there is room for just doubt whether there is even as much difference as this. Now it cannot be reasonably supposed that as much as seventy-two *per centum* of the Morphine is lost, in the process for its separation from Opium, and therefore it is altogether probable, *a priori*, that there is another principle, upon which its virtues may in part depend, more especially as the Strychnos Nux-Vomica, and several species of Cinchona, are now well known to contain two principles, possessing medicinal powers, similar in kind, and differing mainly in degree. Nothing else beside Narcotine, and another very doubtful substance, called extractive matter, having, as is said, an intensely bitter taste, has yet been obtained from Opium, which can reasonably be suspected of contributing to its remedial effects.

For the purpose of ascertaining the precise powers of Narcotine, I have recently instituted a course of experiments, fourteen in number, upon healthy subjects it is true;* but they have afforded results much more satisfactory, and without doubt much more analagous to what will be its effect in disease, than could possibly be obtained from experiments upon brute animals, and especially upon those which have suffered the lesion of a ligature upon the œsophagus, immediately after being forced to swallow the agent whose powers are to be ascertained. As a detail of the experiments themselves, would probably be incompatible with this Journal of Science, it will be reserved for some journal exclusively medical, and this communication will contain only a summary of the results obtained by the experiments in question, preceded by a few definitions, which are necessary to show the precise acceptations in which I employ the terms that will be applied to the several operations of the Narcotine.

In regard to the subjects of my experiments, it is not necessary to make any further statements, than barely to say, that one was a young physician belonging to the State of Massachusetts—that another belonged to the State of New-York—that two others belonged to Connecticut—and that I was myself the fifth subject. The four gentlemen, above mentioned, happened to be in New Haven together, and at leisure for about a week, and they therefore volunteered their assistance in this business, entering into it with a degree of interest and zeal, which fully evinced that with them their profession is not viewed as a mere trade, to be followed only for the purpose of obtaining a support, or as a means of acquiring wealth, but is rather esteemed a liberal art, which they cultivate with much more generous and honorable motives. It may be proper to state in this place that I once made a series of experiments of the same sort, upon the Sulphate of

* It is altogether probable that the state of health may be taken as a medium state of susceptibility to the influence of this article. Under certain circumstances much more may be required, and under certain other circumstances, much less, in order to produce given effects.

Morphine; and that a subsequent employment of that article in my medical practice, has abundantly confirmed all the conclusions to which I then arrived—and it is true, has led to some others, which could not be obtained upon a healthy subject. The result of my experiments upon the Sulphate of Morphine, and of my subsequent observations upon its operation in disease, will be subjoined to this paper on Narcotine, as in some instances my descriptions of the effects of each, have necessarily been comparative.

*Definitions.* A *narcotic* operation consists of four parts, stages, or degrees, viz.

1st. An *antirritant* stage, in which morbid irritability andirritation, and irritative action generally—morbid sensibility, restlessness and jactitation, (when connected with a non-phlogistic, or a positively atonic condition of the system,) are allayed;

2nd. An *anodyne* stage, in which pain, (when connected with a non-phlogistic, or a positively atonic condition of the system,) is relieved;

3d. A *soporific* stage in which sleep is produced; and

4th. *Ultimate narcosis*; in which there is vertigo, headache, faintness, dimness or imperfection of vision, nausea, and retching, epigastric uneasiness, small and irregular pulse, cold extremities, cold, clammy, or slippery sweats, delirium, stupor, convulsions (either common, epileptic, or tetanic), coma, and death. I am confident, from multiplied observations, that all *narcotics* are necessarily *stimulants*.

A state of prostration, (not of exhaustion or debility, as is commonly, but erroneously supposed,) sometimes takes place, as an indirect effect, and rather a remote consequence, of a single dose of certain *narcotics*, too large for the susceptibility of the patient. This state is characterized by vertigo, nausea and vomiting on motion, and headache and faintness. Although these symptoms constitute a part of what I have described as *ultimate narcosis* takes place while a patient is under the fullest operation of the *narcotic* agent, and this sort of prostration takes place only after the direct effects of the *narcotic* agent have passed off, and it is rather a sequel than a direct effect of such an agent.

Different *narcotics* vary very much in the relative degree of each of these states or stages of a *narcotic* operation, which they respectively produce. Each state or stage of a pure *narcotic* operation, may be considered as a strictly sedative operation.

A *nervine* operation consists exclusively of four states, stages, or degrees, viz.

1st. A moderate antirritant stage, indicated by more or less relief of the same symptoms, that are obviated by the first degree of a *narcotic* operation. I do not suppose that the *antirritant* effects of a *nervine* are identical with the *antirritant* effects of a *narcotic*;—they appear to constitute distinct sorts of *antirritant* effects;

2d. The production of a peculiar calm, placid, and pleasurable sensation;

3d. The production of a peculiar preternatural wakefulness; and

4th. The production of more or less positive exhilaration, sometimes amounting to delirium.

Different *nervines* also vary very much in the different relative degree of each of these states or stages of a *nervine* operation, which they respectively produce; and many are altogether incapable of producing the fourth state, or stage in any appreciable degree.

Pure *nervines* may be pushed to any extent whatever, within the capacity of the stomach to contain, without producing a single individual of those symptoms which I have detailed under the denomination of *ultimate narcosis*, and without the least increase of the vital energies generally, or of the strength of arterial action, which is a test always adequate to the perfect distinction of pure *nervines* from pure *narcotics*, and pure *stimulants*.

It is very common to confound a *nervine* operation with a *stimulant* one; but they are perfectly distinct. All the parts of a *nervine* operation (as I have just said) may be produced without any increase of the vital energies, and without any increase of the strength of arterial action. Indeed, I have very often seen the fullest *nervine* operation connected with an extreme reduction of all the vital energies, and with such a diminution of the strength of arterial action, that the pulse could scarcely be felt.

A *stimulant* operation consists exclusively in a quickly diffused, and transient increase of the vital energies generally, and a similar increase of the strength of arterial action. *Stimulants* usually diminish atonic morbid frequency of the pulse; but, in perfect health, they usually, (though not invariably) increase the frequency a few beats. *Stimulants* also commonly diminish, in a slight degree, both morbid irritability and irritation, and irritative action generally; morbid sensibility and sensation; morbid mobility, restlessness, and jactitation; but they do this in a less degree even than the *nervines*, and still less than the efficient *narcotics*, and, as I think, doubtless in a manner different from either. Pure *stimulants* never produce the least trace of the last three states or stages of a *nervine* operation, nor a single symptom of what constitutes *ultimate narcosis*, with the occasional exception of nausea and vomiting, and perhaps headache, from the mere irritation of excessive doses or quantities; nor do they ever produce any condition at all analagous to the secondary and rather remote sort of *prostration*, which, I have already mentioned, as sometimes the sequel of too large a single dose of certain *narcotics*. These circumstances afford absolute tests of pure stimulant powers.

It must be remarked that *narcotic* and *nervine* powers are principally exerted upon the nervous system, while *stimulant* powers are mainly exerted upon the sanguiferous system; and, particularly, that the first three states or stages of a *narcotic* operation—the whole states or stages of a *nervine* operation—and a perfect stimulant operation, are by no means incompatible with each other. Thus, for example, a full and complete *antirritant* operation, as produced by Opium—a perfect *anodyne*, and a prominent *soporific* effect may take place at one and the same time, with a most decided increase of the vital energies generally, and of the strength of arterial action; and either or both of these operations may or may not be accompanied at one and the same time, with all the states or stages of *nervine* operation. *Sedative* effects, then, are by no means, incompatible with *stimulant* effects. What is called *ultimate narcosis*, at least in any prominent degree, does in fact seem to be incompatible, either with positive *nervine* or *stimulant* effects.

The conjunction, at one and the same time, of full *stimulant* effects, with the three first states or stages of a *narcotic* operation, may be witnessed in a prominent degree by the use of moderate and uniform doses of Opium and Alcohol, at regular and short intervals, for a certain length of time, in any case to which both are appropriate remedies.

That *narcotic, nervine,* and *stimulant* operations, as here defined, are perfectly distinct operations, is abundantly proved by the fact that there are numerous articles which possess each individually, without any trace of the others; and the circumstance that two of these groups of effects, or even the whole three, are not unfrequently produced by the same articles, more or less proves their identity, than the circumstance that Tobacco is both *narcotic* and *cathartic*, proves that these two effects are identical.

### *Results of Experiments with Narcotine.*

1st. In the same quantities, Narcotine is far less operative upon the human system than the Sulphate of Morphine, and even less so than Opium.

2d. Narcotine possesser the same degree of activity, when given pure and in substance, as in any other way tried, its virtues not being either enhanced or diminished by solution in oil, or in dilute Acetic Acid.

3d. From two to five grains of Narcotine appear to constitute a medium full dose, where only a single dose is to be taken; i. e. such a dose as will just fall short of producing any disagreeable effects in a person of ordinary susceptibility.

4th. One grain of Narcotine appears to constitute a medium moderate dose, to be repeated at regular and short intervals;—and three hours appear to constitute a medium suitable period of repetition for such a dose, for a person of ordinary susceptibility.

5th. Narcotine is much slower, though it is less prominent in its effects than the Sulphate of Morphine. The period required for the first manifestation of the several effects of Narcotine, is intermediate between that required for the effects of the sulphate of Morphine, and that required for the effects of Opium; and the period of their duration is intermediate between that of the effects of opium and that of the effects of the Sulphate of Morphine.*

6th. Narcotine appears to be more or less *nervine*. The general *nervine* operation of Narcotine, I think is decidedly less than that produced by sulphate of Morphine, but I am not certain that it is less than

* It is remarkable that Morphine, which is much more speedy in its operation, appears to be more permanent in its effects even than Opium.

that of Opium. Narcotine does not appear to produce any of that preternatural watchfulness, which so often results both from sulphate of Morphine and from Opium. From the circumstance that Sulphate of Morphine possesses this last-mentioned property in an eminent degree, and that Narcotine is destitute of it, it follows that the same property of Opium is dependent upon its Morphine, and not upon its Narcotine. In short the quality of the nervine operation of Narcotine is considerably different from that of Morphine, and consequently more or less different from that of Opium.

7th. Narcotine appears to be considerably diaphoretic, and it commonly produces more or less itching of the whole surface, which is first perceived, and is most considerable, on the inside of the thighs and about the nose.

8th. Narcotine is most prominently and most decidedly narcotic.

As soon as it begins to produce a decided effect upon the system, it occasions a very peculiar expression of the countenance, which is more easily recollected than described. There seems to be a peculiar elongation of all the features, and a kind of lateral shrinking of the whole face, which, together with the effect upon the eyes, and particularly the contraction of the pupils, more unequivocally indicates the operation of a *narcotic*, than any expression of the countenance which is produced within my knowledge by any other agent. While in the early stages of its operation, and before my family knew any thing of the experiments, one individual of them after another, noticed this expression, and made remarks upon it. One said I appeared as if about to be attacked with some acute disease—another inquired if I had got *Sick headache* to which I am subject) and each made some comment. Similar remarks were made to the other gentleman. One was met in the street by another physician, who immediately pronounced that he was under the influence of some active *narcotic*.

Narcotine very materially and very greatly reduces the frequency of the pulse; it allays very effectually certain sorts of cough; it occasions indistinct vision, or the sensation of a blur before the eyes; and when a person is strongly under its influence, it occasions a contraction of the pupils. It produces also a sensation of dryness and clamminess in the mouth, though it appears sufficiently moist to the eye; and it produces not only a change in the sound of the voice (while a person is under its influence), but likewise very considerable hoarseness. These effects occasionally take place quite early in this operation. It produces not only a considerable diminution of the natural excretion by the renes, but also a deficiency of contractile power, or torpor of the bladder.

Whether Narcotine is constipating or not, may perhaps be considered as somewhat uncertain, but it is most probable that it is so. While experimenting upon it, one of the gentlemen had a regular and daily alvine discharge; but on account of a much greater susceptibility, he took considerably less of it than the other gentlemen. On the contrary, one gentleman had none for three days, while taking it; and another gentleman had none for five and I think six days, while under its use. Since the completion of the experiments, I have known it taken twice for a moderate diarrhœa, with perfect relief of the disease. It may, however, possess the power of relieving diarrhœa, i. e. of obviating morbid irritability, and irritative action of those muscular fibres, which produce the peristalic motion of the intestines, without being liable to produce constipation; i. e. to lessen healthy excitability, and natural peristaltic motion. The resin of the *Zanthorrœa hastilis* operates in this manner.

The *antirritant* effects of Narcotine appear to be greater in proportion to its other powers, than the *antirritant* effects of Opium; and also, as appears to me, had the *antirritant* effects even of the Sulphate of Morphine. Its great power of diminishing the frequency of the pulse, seems to indicate this, as well as its power of allaying certain sorts of cough. No opportunity has occurred of testing the *anodyne* powers of narcotine. It will be obvious that this cannot be determined upon a healthy subject. The *soporific* effects of Narcotine appear to be considerably greater, in proportion to its other powers, than the *soporific* effects of Sulphate of Morphine, or than the *soporific* effects of Opium. The sleep produced by it, even when taken in a moderately excessive dose, is peculiarly calm, light, placid and easy; and even when it is the most intense, the subject of its influence is easily roused: and by voluntary bodily motion and exertion, he can easily keep himself awake, and apparently very much diminish its general influence upon his system—and yet, as soon as he sits down and remains quiet for a short time, its full influence speedily returns. During the deepest sleep produced by Narcotine, the respiration is light and easy, like that of a person in health, who has been some time perfectly at rest.

When the subject of its influence awakes spontaneously from the sleep which it produces he feels no heaviness, and nothing unnatural, but much as awakening in the morning from an ordinary night's rest, except that he has a slight sensation of dryness and clamminess in the mouth, a considerable hoarseness, diminished renal secretion, and diminished contractility of the bladder.

In a moderately excessive dose, in relation to the susceptibility of the system, Narcotine produces a mazy and confused state of the head, vertigo, nausea, and vomiting. Too large a quantity in the twenty-four hours, operates in the same manner. But the effects of a moderately excessive dose of Narcotine, are much less disagreeable than the effects of an excessive dose of the Sulphate of Morphine, or of Opium. The mazy and confused state of the head, and even the vertigo which it produces, are attended with a decidedly pleasurable state of the feelings; and even the nausea and vomiting which it occasions, are by no means distressing, and are far less unpleasant than the similar symptoms produced by Sulphate of Morphine, and by Opium. The nausea and vomiting which a moderately excessive dose of Narcotine produces, begin almost instantaneously, and terminate as suddenly; and, in a very short time, no sensations remain, which indicate that nausea and vomiting have occurred at all,—there is no violent straining, no weakness, soreness, or stiffness afterwards.

According to Magendie, and others, the *ultimate narcosis* of Narcotine is made up of the following symptoms, viz.: signs of fright; backward movements, with incapacity of advancing; frothing at the mouth; agitating or tremulous convulsions of the jaws; general convulsions of the common sort; tetanic spasms of the extensor muscles of the neck, throwing the head backwards upon the spine; a stupor, in which the eyes remain open, but from which the subject cannot be roused, and under which he dies in the course of twenty-four hours. These last seem to be the only effects of Narcotine that have been heretofore fairly determined, either in Europe or in this country, at least within my knowledge. It is obvious that these could not be verified on the human subject, nor is it necessary to know them, for the therapeutic application of this agent, in the treatment of human diseases. Magendie says that these effects are similar to those produced by fatal doses of Camphor; and what is remarkable, he pronounces them to be mere *stimulant* effects, and not at all indicative of any *narcotic* powers! I wish that Magendie had furnished us with his precise views of the nature of an *ultimate narcotic* operation, for I cannot conceive of a purer one than is indicated by this aggregate of symptoms. I venture to assert on the one hand, without the least fear of contradiction, that if no articles which are capable of producing effects of this general character are suffered to retain their place among the *narcotics*, our catalogue of this class of agents will become extremely meagre; and, on the other, that there is not a pure and unequivocal stimulant that is capable of producing any such symptoms.

9th. Narcotine appears to be altogether destitute of all *stimulant* powers, whether it is given in single full doses, or in moderate and uniform doses, at regular and short intervals. My attention, during the whole of my experiments, was particularly turned to the question of its *stimulant* operation; and in no case, while under its influence, was there the least perceptible increase of the vital energies, or of the strength of arterial action, or even of animal heat; nor was there any sensation of fullness of throbbing in the head; nor, indeed, did any symptom whatever occur, which could by any means be construed into an effect of this sort. On the contrary, there was invariably a great reduction in the frequency of the pulse, in two cases as great as twenty-six beats in a minute, and in none less than eighteen in the same time. In some of the cases, there was a decided diminution, both in the force of the pulsation and the fullness of the artery, and probably more or less in all, though in some it was so inconsiderable as to be of very little consequence. These effects, I repeat, occurred equally, whether the agent was given in single full doses, or in moderate and uniform doses, at regular and short intervals, and whether taken in substance or dissolved in dilute acetic acid, or in olive oil, the power of producing preternatural watchfulness, even were it possessed by Narcotine, would not indicate any *stimulant* properties, but rather mere *nervine* ones, which are entirely distinct. The power of producing vertigo, headache, faintness, nausea, vomiting, irregular pulse, cold extremities, etc. is not the result of a *stimulant* operation, but of a *narcotine* one; and both Morphine and Narcotine are capable of producing all of these last effects, though Morphine more eminently than Narcotine. If these results can be considered as at all correct (and I cannot discover where there is any possible source of fallacy), the futility of what is called *denarcotizing* Opium, as a means of improving its medicinal operations, will at once be manifest. However, as the effects of Morphine and Narcotine differ considerably, not only from each other, but also from Opium, it is undoubtedly useful to have each of these proximate principles in a separate state, that we may be able the more accurately to adapt our remedial agents to the peculiar circumstances of a given case.

What quantity *per centum.* of Narcotine is contained in Opium, I know not.* As in a given quantity it is less active than Morphine, and even less so than Opium itself, no quantity of it, however large, will account for the full effects of Opium, upon the supposition that we are correct as to the proportion of Morphine. But I do not esteem it by any means impossible, that the *bitter principle* already referred to, as being called by the vague name of *extractive* (if indeed there is any such distinct principle), or perhaps some other part of this complex drug, may yet be found to contribute something to its medicinal effects. —*Bost. Med. and Surg. Jour.*

4. *Test for Mercury and its Salts.*—"The most delicate, perhaps, is that proposed by Mr. Sylvester. A drop of the liquid supposed to contain it is to be placed on a piece of gold leaf, or any piece of solid gold, and the point of a nail or pen-knife, or of any small piece of iron or zinc placed in contact with the moistened surface; if any mercury be present, the gold will immediately become white, where it is touched by the other metal, uniting with the mercury and forming a solid amalgum, which retains its white color after the fluid has been wiped off.

Put a piece of copper into a solution of any salt of mercury; and the copper will be dissolved, combining with the oxygen of the oxide of mercury and the acid with which it was previously united, while an equivalent quantity of metalic mercury will be precipitated.

Add a solution of potassa to a solution of salt of mercury; oxide of mercury will be immediately precipitated. If the mercury in the liquid shall have been in the form of a protoxide, the precipitate will be of a dark color, but when it contains the peroxide alone, the precipitate has sometimes a reddish color, but is generally yellow, the peroxide disengaged combining with a portion of water and forming a hydrate. Collect the precipitate on a filter, dry it, and expose it to heat at the bottom of a small test tube over a spirit lamp, when globules of metallic mercury will be seen.

* It has been said (upon how good authority I cannot decide,), that Opium usually contains about twice as much Narcotine as Morphine.

Put a small quantity of a solution of a salt of the protoxide of mercury into a glass and fill it up with lime water; the protoxide of mercury will be immediately thrown down and give a dark color to the liquid.

Into another glass put a similar quantity of a solution of a salt of the peroxide of mercury, and add lime-water as before; the solution will become of a yellow color from the separation of peroxide of mercury in combination with water.

Add some sulphuret of ammonia, or pass a stream of sulphuretted hydrogen gas through a diluted solution of mercurial salt, a copious black precipitate will be thrown down of sulphuret of mercury, probably in combination with water.

Add a solution of the muriate of tin (muriate of the protoxide) to a solution of the salt of protoxide of mercury; a copious ash-grey colored precipitate will immediately appear, consisting principally of metallic mercury, the oxide of tin combining with the oxygen with which the mercury was previously united, and passing to a higher state of oxidation.

A solution of the ferrocyanate of potassa gives a white precipitate with salts of mercury; muriatic acid and solutions of the muriates give a white precipitate, with salts of the protoxide, composed of the chloride of mercury.

All the salts of mercury are completely decomposed or volatilized by exposure to a dull red heat."—*Reid's Elements of Practical Chemistry.*

5. *Facts relating to Hydrophobia.*—Silliman's Journal of Science for October, just received, like all the previous numbers which we have read, is replete whith valuable and interesting matter. Among other articles which have attracted our attention, is a letter from the Rev. William Case, of Chester, Conn. to the Editor, giving the particulars of four remarkable cases resembling hydrophobia which occurred in that township, and which favor the idea, not only that the canine virus may take effect many years after it has been received into the system, but that, contrary to the opinions usually entertained, it may be communicated from one person to another. We subjoin so much of the letter as relates to these particular cases.

Chester, Oct. 14, 1832.

*Dear Sir,*—In compliance with your request, I have endeavored to collect the facts, respecting the supposed cases of hydrophobia in this place, and the transmission of the poison from one human being to another. I ought, perhaps, to state, that most of the physicians consulted in the three cases of supposed transmission from one human being to another, attributed their death to some other disease, such as might be supposed to be attended by symptoms similar to those which characterize this malady. It is believed, however, that the material facts in these cases, were never fully made known to these gentlemen. Neighbors and attendants, in whose possession they were, feared, perhaps, to disclose them.

In 1807, W. C.* was bitten and wounded by a mad dog, when at the age of eleven years, and on his way home from school. After biting this youth, the dog was confined in a small apartment in his owner's house, where he was seen by many persons, and where he exhibited all the symptoms of hydrophobia. A person, in company with others, with a gun in hand, ascended the chamber stairs, displaced a part of the floor, and through the aperture shot and killed the dog. These facts are attested by a man who was an eye witness, and they are corroborated by many others.

At the time of the bite, in 1807, W. C. is said to have had a feeble constitution, but it is testified on all hands, that he grew up without sickness. It is said, he conducted strangely, by turns, sometime before his last sickness. The disease appeared in him fifteen years after the bite, and was preceded by mental irregularity. He had a short season of strange excitement, during public worship on the Sabbath. At a neighbor's house, the next day, he suddenly jumped, screamed, broke windows, and ran out at the door, with great nimbleness of foot. He soon became quiet and returned, and when his friends remonstrated with him for this conduct, he said he could not avoid doing thus, for he had been bitten by a mad dog. During the progress of the disease, he gnashed his teeth;† discharged large quantities of saliva, had distressing spasms, and was set on biting every body and every thing. The pillow cases through which he made holes, by taking them in his teeth and shaking them, are now to be seen. He spit on persons who came in, and on all parts of the room. He was averse to swallowing any thing. He watched for opportunities to bite persons, and if he could bite any one, it seemed to afford him pleasure, and was followed by laughing. He lived after his attack, fourteen or fifteen days. It required four or five able men to attend upon him. He died Sept 1st. 1822, aged twenty-six years.

L. T. C., S. W. H. and C. C. a brother of W. C. were bitten while attending on him in his last sickness. The bite in these three cases drew fresh blood from the hand or wrist, and this fact is attested by many witnesses. These three cases were preceded by mental anxie-

* The rabid dog which bit W. C., was seen, when he was bitten by another dog supposed to be rabid. I had the facts from a gentleman now residing in Ohio, and as nearly as I can recollect, they are as follow:—A strange dog crossed the orchard adjoining the house, and was seen, without provocation, to seize and bite the dog which bit W. C. The gentleman's daughter, then a small girl, now the widow of C. C., was in the orchard at the time, and her mother, seeing the dog in the act of biting the other, and being alarmed, called her in. The circumstance she well remembers, and says the bitten dog fled into her father's house, instead of his owner's, not more than four rods distant, and ran under the bed. The stray dog afterwards appeared in front of the house and sat down. The gentleman pointed out the spot, described his appearance, and said he had not a doubt of his being mad. If I do not misremember, he was destroyed. The bitten dog bit W. C. between two and three weeks after he received his own bite.

† It is stated also that he howled and growled; but it is easy to suppose that the imagination may convert groans and shrieks of distress into imitative sounds.

ty, and followed by spasms, delirium, and lucid intervals. The first spasms were of short duration, and attended by jumping, hopping, and screaming. Successive spasms continued longer and became more severe. The eyes of all assumed a glassy and watery appearance. What I have termed discharge of saliva, was, in all these cases, called frothing at the mouth.

L. T. C. was a strong athletic man, he was bitten in 1822, by W. C. and after a sickness of two weeks, died March 13, 1826, aged 32 years. Some weeks before his confinement, he exhibited symptoms of mental aberration.* He would hop backward and forwards, and talk incoherently, for a few minutes, and then say, he was sorry he conducted so, but he could not avoid it. His attendants say, the taking of water or drink made him rave. A spectator observes, that he sometimes called for drinks, when it seemed as if he thought they would be refreshing, and do him good; but no sooner had he filled his mouth with the fluid, than he would spirt it in the face of him who offered it, and decline to swallow the drink. So strong was his aversion to swallowing, that a near relative questions whether he could swallow. Another says, that drinks were sometimes forced down; but he shuddered at swallowing. With his mouth he seized by the arm, a person attending upon him, and through thick clothing, left upon his flesh the print of his teeth. He immediately said, "Now I have hurt you, and I am sorry; but I could not avoid it, I must either die myself or bite you." If he had not been confined, says one, I have no doubt he would have bitten every person in the room. A part of the time it required seven able men to keep him to his bed and in his chamber. It is said that he was not known to walk to, or from the bed; but always leaped off and upon the floor; he would whirl suddenly around, and shift his position on the bed, and sit on his feet, and the by-standers imagined that he imitated the motions and the barking of a dog. He frothed at the mouth, and ran out his tongue. He spirted drinks into the face of his attendants, spit on them, on every body and every thing, and all over the room.† His efforts in spasms exhibited such strength as literally to frighten his attendants.

S. W. H. sickened and died August 10, 1807, aged 30 years. He was sick five days, although he had been for years subject to epilepsy, yet there was no appearance of this disease in his last sickness. He was bitten by W. C. in 1822, and carried the scar of the bite to his grave. At the time, there was no general apprehension of hydrophobia, nor any excitement on the subject. It is not known that the fact of his bite

* It is not supposed that there was any proper delirium, but merely that degree of aberration which might be supposed to arise from the violent paroxysms of the disease in a very strong muscular subject. L. T. C. and C. C. particularly, were very powerful men, and C. C. was unrivalled as a wrestler.

† The prevailing popular impressions on this subject are well known; perhaps it is not surprising that some of the distressing and various appearances in hydrophobic patients should be attributed, by the terrified beholders, to a specific canine influence. Medical men do not admit the genuineness of these appearances.

was mentioned to his physician. His disease was pronounced to be something else. Whatever it might have been, it was preceded by mental anxiety, and attended by the following symptoms. He at first inclined to wander from his house, and gave indications of mental aberration, and of a great dread of water. He resided near a small lake, and before being confined to his room, requested his companion to keep him from the water, and be sure and not let him get into that lake; if he did it would kill him. He is represented as having had, in the first stages of his disease, a constant dread of water, but six hours before he died, he called for some, and drank of it. Before his spasms commenced, he entreated his wife, that if he should be as W. C. was, she would procure some strong man to take care of him, and be careful to get some one that would make him mind, lest he should hurt others. During one of his spasms, he bit his tongue, and loosened a piece of it. He requested his wife to take the scissors and cut it out, but to take care and not get any of the blood on her. He would take drinks to oblige his friends and attendants, but they say he always swallowed with a convulsive effort, such as they cannot describe. The disposition to wander continued to the last. He tried various expedients to be released, and to escape from the house. He would grin, and fix his glaring eyes on his attendants, when near him, in such a manner as to make them guard against being bitten. He would take a handkerchief and bed clothes in his teeth, and bite and shake them. He is represented as of a peaceable disposition, and some of his friends think that he made great efforts to curb the disposition to bite.

The death of L. T. C. and S. W. H. left on the minds of the community an impression of mystery. There was a general feeling that the cases were very singular. Individuals, who witnessed their sickness, and were acquainted with the facts above mentioned, firmly believed that they died of hydrophobia, but no professional man had encouraged that belief, or laid stress on the facts which supported it, and the friends, for obvious reasons, were not forward to express it.

Things remained thus, till C. C. a brother of W. C. sickened and died March 24, 1828, aged thirty-seven years. He was sick but eleven days. He was a strong laboring man, and had never before been subject to sickeess. After attending a meeting in the evening, he retired to rest, and slept as usual. About midnight he suddenly sprang from bed, ran undressed into the street, screaming so loudly as to alarm his neighbors. From this first decisive appearance of his disease, remedies seemed not even to abate his distressing symptoms. Nor was his malady suspected by his attending physician, till the third or fourth day. On my making enquiries of a parishioner in regard to the sick man, he said to me, "He is better. Do you know what the people say about C.? they say he was bitten by his brother W. who died of the hydrophobia, and they can prove it, and they know he is mad." I immediately made enquiries, and became satisfied that this was not mere talk. I saw, the next day, his attending physician, and having mentioned the facts to him, I asked if he had suspected the nature of the disease. He replied no. I then enquired if he would not examine his authorities, and look for the symptoms of the disease, with sole reference to them. His next visit to the sick man resulted in his conviction that his disease was hydrophobia. The physician had told the attendants, that if they would preserve some of the saliva, and inoculate a dog with it, he would become rabid in a certain number of days. They had carefully taken up on a woollen string and dropped into a phial a quantity of this and corked the phial. They had selected the victim and the place of his confinement, and were holding the corked phial in the hand, when the man was seized with his last spasm. The phial was suddenly dropped, and all search for it in future proved ineffectual—a circumstance deeply to be regretted, for had the experiment been made, the result would probably have removed from every mind, capable of appreciating it, all doubt respecting the sick man's disease. The following are some of the facts as stated to me by his physician, and corroborated by his widow and those who attended him. The sight of water produced a recurrence of distressing spasms, or in the language of attendants made him rave. In the intervals of spasms he was rational. In one he requested his father-in-law to remove and hide his razors, for he did not know what he might be led to do in his turns. In another, he gave this caution to his wife. "I wish you to keep away from me when I have these turns; I know not why it is, but I want to bite, and fear I shall bite you." His attendants think he strove to curb the disposition to bite. It was however very evident. A neighbor one evening entered the room. On seeing him, he immediately said in a pleasant way, "How do you do, Mr. B——? I am glad to see you. Come here, I want to shake hands with you." The neighbor approached and extended his hand. The sick man seized it instantly, and with a convulsive spring, rose from the lying posture and drew it to his mouth. The attendants who stood near, and expected this result of shaking hands, instantly seized Mr. B——, and forced him from the sick man's grasp before he was bitten. He talked much about biting, and the attendants, as usual in such cases, imagined that he growled, snapped and barked like a dog. The shaking of pillows and bed clothes in his teeth was a frequent exercise. His eyes were glassy and watery. He spit much the night after he left the bed, and during his sickness. He spit on all parts of the room, and watched the opportunity to spit on persons who came into it. During his sickness, and especially the night before his death, he screamed and hallooed dreadfully.

On the supposition that these are to be regarded as cases of real hydrophobia, the facts will stand thus.

1. W. C., first victim, bitten by a rabid dog, in 1807. Sick fifteen days. Died fifteen years after the bite, in 1822, aged 26 years.

2. L. T. C., second victim, bitten by W. C. in 1822. Sick fourteen days. Died short of four years after the bite, in 1826, aged 32 years.

3. S. W. H., third victim, bitten by W. C in 1822. Sick five days. Died five years after the bite in 1827, aged 30 years.

4. C. C., fourth victim, bitten by W. C. in 1822. Sick eleven days. Died short of six years after the bite, in 1828, aged 37 years.

These facts are well established, and they preclude the necessity, if not the propriety of referring the symptoms of their disease to what is sometimes termed spontaneous hydrophobia. Nor did the previous habits of life in a majority of the patients, at all favor such a reference of their symptoms.

C. C. was a constant spectator of his brother W. C. in his last sickness. They died in the same house. C. C. also watched with L. T. C. and S. W. H., and repeatedly told his wife, as she now affirms, that they were sick just as his brother was. A sensible man who witnessed three of the cases says the persons were sick alike as nearly as the difference of natural dispositions and habits of life would permit. Different individuals, who observed attentively two or three of the cases, bear the same testimony in such numbers, as to include in it all the four. With persons at all acquainted with the facts and cases, it is now the general opinion that the disease was hydrophobia.

All four dreaded confinement, and exhibited great quickness of apprehension in regard to persons, and circumstances which surrounded them. There was uncommon agility and sprightliness in their motions, and an evident display of cunning, especially in their efforts to escape and bite persons. Till within about two days of the termination of the disease, the patients were not only inclined to escape, but a primary object of the intended escape, was to have opportunity to run. It is the opinion of the attendants, that if they could have escaped, they would have run till they dropped down dead. One among other things, pretended to have business at a place sixteen miles distant. He plead with his attendants to let him place his feet on the door steps, and assured them that if he might do it, he would run so that no man could overtake him till he had reached the place.

The fears of all were that they would injure others. One was, at times, afraid of receiving injury from others; but this fact, it is believed, may be explained, by a reference to circumstances peculiar to himself. All that is claimed, however, is that there was a general concurrence in the class of symptoms to which allusion has been made. From momentary agitations, connected with slight mental aberration, the spasms gradually increased, in duration and force to the last. One attendant says the strength of the patient seemed to increase during the spasms, and he finally sunk under them. This man's services have always been sought, and freely rendered in extreme sickness. He affirms that he never witnessed cases resembling these. In attending on the last individual, he covered with rags spots of fractured skin on his hands, least the saliva of the patient should reach them. Another procured and constantly wore, a pair of stout gloves. An attendant in one of the cases, says, that twelve hours' services at a time, was as much as he could endure, and that others were obliged frequently to change, and retire from the scene, and suspend the efforts required of them when present.

Many testify that there was in all the cases, during the spasms or spells, as termed, an uncommon scent from the patient's breath. This was observed in every case by numbers. Some designate it by the epithet strong; others say they never experienced the like before, nor since, and they cannot describe it. All seem to remember it as perfectly distinct in this character. One person, in endeavoring to convey an idea of it, said it resembled that of cats and dogs when fighting. This smell was not perceived, except at the times when the patient raved, or had spells and frothed at the mouth.

It is the opinion of attendants, that the patients were literally stiffened during the spasms, and that in the latter stages of the disease, they might have been raised up erect by the application of force to the head, without any bending of the body. The corpses were stiff immediately after dissolution, and the jaws set so as to require no muffler. On them, and near the surface of the skin, blue spots appeared, which some mistook for indications of mortification. One was kept four days, and on this body the spots wholly disappeared, and it underwent no other visible change. It is stated that the spots appeared as soon as the patient was supposed to be struck with death, and that when they disappeared, they left the skin slightly affected, and of a greenish hue. It is said the corpses had a strange appearance, the countenance resembling that of a living person in health when cold. Some designate this appearance by the epithet blue.

P. S. An intelligent medical man, who has heard the above statement, and conversed largely with witnesses, believes these cases to have been hydrophobia. Two of them have been attributed by others to delirium tremens, but the previous habits of the patients did not favor that idea; none of the four were addicted to the use of ardent spirits, and one of them had a constitutional aversion to distilled liquors.

The above cases possess so many points of resemblance to hydrophobia, that it may appear unreasonable to deny them to have been of that character; but at the same time, it cannot be denied, that there are many circumstances which leave it very questionable. 1. In the first case, the great length of time which intervened between the bite and the attack of the disease, is, in itself a suspicious circumstance. Numerous cases of supposed hydrophobia are indeed recorded, in which a similar interval has occurred, and if the poison may lie dormant in the system for one year, unaffected by the action of the absorbents, it is not easy to conceive why it may not for an indefinite length of time. The hydrophobic character of these diseases, however, has been generally doubted by the best medical writers, and these cases have seldom been attended with such evidences of authenticity as to place it beyond doubt; until the fact therefore be completely established that the virus may lie dormant in the system for years, and eventually produce the disease, humanity requires that the belief should not be countenanced.

2. The symptoms do not very accurately correspond with those which are usually described as characteristic of this disease. Popular belief, it is true, ascribes to such patients all the symptoms which attend rabies in animals; but in those cases that have been recorded by medical men, neither the disposition to bite, nor the mental alienation have been at all prominent symptoms. That such patients might accidentally bite their attendants in the agony of the convulsive paroxysms to which they are liable, may be easily imagined; but they are generally inoffensive and preserve the full possession of their intellectual faculties until the last moment of their lives. Excessive nervous agitation is the prominent symptom of the disease.

3. Two of these patients appear to have been constitutionally liable to nervous affections—one of them having been affected with derangement some weeks before his confinement, and the other having been for years subject to epilepsy.

4. The duration of the disease is not very consistent with the speedy termination of hydrophobia—two of them being sick two weeks, and the other eleven days before death.

5. The aversion to water does not appear to have been as prominent a symptom as it generally is in hydrophobia. There is no mention of it in relation to the first individual—the second and third took liquor into their mouths, and one of them called for water and drank it a few hours before his death.

6. There is evidently inaccuracy in the details of these cases, arising from defect of memory and the influence of the imagination in those from whom the facts were collected. Implicit confidence can seldom be reposed in the reports of disease, even when drawn up, at the time, by persons whose education and habits of observation might be supposed peculiarly to qualify them for the task, still less when drawn up at the distance of years, by persons who are unaccustomed to discriminate the symptoms of diseases. F.

6. *Aneurism from Wound of the Brachial Artery in Venesection.*—"Four forms of aneurism may arise in the bend of the arm from a wound of the brachial artery (sometimes the radial) in venesection, aneurismal varix, varicose aneurism, diffused and circumscribed false aneurism. Respecting the treatment of the last two, considerable difference of opinion exists among practical surgeons, some contend that both require an operation, while others maintain their curability by compression.

The advocates for the operation have not as yet decided upon the precise situation where the vessel should be secured, nor upon the mode of securing it, some apply a ligature upon the vessel above the aneurism, and depend upon the absorbents for the removal of the tumor, whilst others open the aneurism, and tie the artery above and below the wound.

The supporters of the mode by compression have not yet agreed either upon the place where the compressing force should be applied, nor upon its degree; some direct violent pressure to be made upon the vessel above the aneurism to obliterate its canal, whilst others are content to apply very slight pressure to the tumor itself.

In the midst of such conflicting opinions, we must appeal to facts, before we can arrive at any thing like a satisfactory conclusion.

Case 1.—*Circumscribed Traumatic Aneurism—Cure by Compression.*—John Miley, æt. 27, applied at Stevens' Hospital on the 11th of October, 1828, with circumscribed aneurism of the brachial artery in the bend of the arm. The tumor was firmly pulsatory, about the size and shape of a pigeon's egg, apparently intimately connected with the artery. It was diminished by pressure, and regained its size on its removal. The radial pulse was weaker on that side than on the other. The integuments presented a small cicatrix. The patient had been bled by a *blacksmith* thirteen days previously. The patient observed "the beating tumor" on the removal of the bandage several days afterwards. The case appeared a fair one for the employment of compression.

The patient was immediately confined to bed, the limb placed upon a pillow, some cathartic mixture prescribed, and sixteen ounces of blood extracted from the opposite arm.

The next day, a thin compress of wetted lint was laid upon the aneurism, and a roller bandage applied from the fingers to the bend of the arm, in the manner recommended by Genga, care being taken that the compression should not extend above the aneurism, the turns of the bandage over the aneurism were very loosely applied. The patient was desired to keep the compress constantly moistened with cold water, and to take a draught containing two drops of tincture of digitalis every sixth hour. Two days subsequently the bandage was opened and re-applied, the draught was continned, and he had sixteen ounces of blood taken from the opposite limb.

On the 7th day, the aneurism felt more solid, and did not diminish so sensibly when compressed. The bandage was re-applied, and a compress of sponge substituted for the lint. The digitalis continued, and other treatment as before.

On the 17th day, the aneurism appeared sensibly diminished, and much more solid; the pulsation in it was less distinct, whilst that at the wrist appeared more full. Bandage continued; the turns which passed over the compress being applied more firmly than before. Pulse 66. Patient is in perfect health, but complains much of the strict antiphlogistic regimen which he is compelled to observe. He was ordered to take a draught containing thirty drops of tincture of digitalis every second hour. In the evening his pulse was down to 38. He did not complain of nausea or giddiness. The draughts were continued. On the 14th day, the digitalis was administered in doses of twenty drops every sixth hour.

On the 20th day, the tumor was much larger than a marble, perfectly hard, and without pulsation. The radial pulse beat firmly, and nearly as full as on the opposite limb. The bandage was re-applied, the compressing force being increased. Digitalis omitted. Antiphlogistic treatment still persevered in.

On the 30th day, there was no trace of the aneurism; the brachial artery could be felt pulsating strongly beneath the cicatrix in the integument, and the radial pulse appeared as full as that of the opposite limb.

On examining the patient eighteen months subsequent to his dismissal from the Hospital, no trace of the aneurism could be detected, the artery pulsated beneath the cicatrix strongly; the pulse at the wrist felt perfectly natural, and he stated that he had not suffered the slightest inconvenience from the arm in pursuing his very laborious occupation."

Case 2.—*Circumscribed Aneurism—Compression—Cure.*—Terence M'Evoy, æt 25, was admitted into Dr. Stevens' Hospital, March 2d, 1829, with circumscribed aneurism at the bend of the arm. The tumor was distinctly circumscribed, fully as large as a turkey's egg, pulsating, uniformly elastic, except at the anterior part, where it felt soft and pulsated most strongly. Pressure on the brachial artery stopped its pulsation but did not diminish its size. An artery of considerable magnitude ran over its anterior surface, but did not seem to be connected with it. The integuments were healthy; an old cicatrix was observable on the centre of the swelling. Nine months previously he had been bled, and, on removing the bandage three days afterwards, he noticed a small pulsating tumor. The treatment so nearly resembles that adopted in the preceding case, that we need not stop to particularize its items. On the 50th day there was no trace of the aneurism. The artery which had been wounded could be felt pulsating strongly beneath the cicatrix at the bend of the arm, and the vessel which ran across the tumor (the radial) appeared perfectly natural.

Case 3.—*Circumscribed Aneurism—Compression—Aneurism rendered diffused—Operation—Cure.*—John Byrne, æt. 35, admitted

February 25th, 1830, with circumscribed aneurism of the brachial artery, produced by wound of the vessel in venesection five weeks previously. The tumor was oval, as large as an orange, adhering to the brachial artery, solid at the base, soft and elastic in the centre. On compression of the brachial artery the tumor lost its pulsation and became softer.

The same treatment was adopted as in the former cases, and, on the eighth day the tumor appeared more solid. The pressure was reapplied with more firmness. He passed a restless night, and on the following morning complained of great pain in the tumor and arm. The aneurism was found to be no longer circumscribed, but to extend along the inner edge of the biceps muscle towards the axilla, for several inches. The whole swelling, particularly the upper part, was soft and fluctuating; the pulsation throughout not very distinct; in short, it was now a case of diffused aneurism. In consultation, it was determined to treat it as a wounded artery. The tumor was fairly opened into, the coagula turned out, the artery tied above and below the wound in it. The ligatures were thrown off on the twelfth day, and on the 30th he left the hospital quite well.

It is manifest from the foregoing cases, that the form of aneurism to which pressure is most applicable, and in which it is most likely to effect a cure, is that of circumscribed false aneurism, unattended by inflammation either of the sac and surrounding parts.

It is equally obvious, that the pressure should be applied to the tumor alone, and not to the artery above, and that the degree of pressure be slight until the diminished pulsation and solidity of the tumor indicate that coagula fill the sac, otherwise the aneurism will become diffused, as took place in the third case. It is probable, if not certain, that the opinion hitherto entertained respecting the obliteration of the artery of the part wounded, is, in the majority of cases, erroneous and without foundation. The pulsation beneath the cicatrix remained strong and natural, after the absorption of the tumor in both cases, and in a case of circumscribed false aneurism in the bend of the arm from bleeding, which Mr. Wilmot cured by pressure, several years ago, in Jervis-street Hospital, the artery continues to pulsate strongly beneath the cicatrix to this day.

The utility of combining Valsalva's mode of treating internal aneurisms with gentle pressure on the tumor itself, was well exemplified in these cases."

7. *Chronic Hydrocephalus cured by Puncturing.*—A child, aged four months, presented a well-marked case of this disease; the largest circumference of the cranium was 18 1-4 inches; the fluctuation was very perceptible at the fontanelles; for when pressure was made upon one, the other was distended and forced out. M. Graefe, compressing the larger fontanelle, plunged a large cataract needle into the smaller one to the depth of the third of an inch. A little viscid fluid escaped, drop by drop; he, therefore, introduced a very fine trocar, and immediately a transparent yellow-brown liquid flowed out freely—

uniform pressure was made on the head as the water escaped. When an ounce and a half had been evacuated, the infant began to faint, and became extremely languid. The canula was, therefore, removed, and a firm bandage put round the head. Stimulants were given, but the child was restless and ill for two days. A similar train of symptoms followed each operation, which was repeated generally at an interval of 12 or 14 days, and sometimes between two and three ounces were discharged at a time; the size of the head sensibly diminished, and the general health improved. The puncturing was repeated eleven times in the year 1829, on the following days—8th, 15th, and 23d of January, 19th of February, 5th and 19th of March, 19th and 27th of April, 5th and 17th of May, 23d of June. The evacuated fluid was thicker and more coagulable towards the end of the cure. The smaller fontanelle and the sutures in the course of time closed; the child became healthy and strong. When ten months old it began to walk; and on the 26th of November, 1830, the child aged two and a half years, was presented well and active to the Society of Medicine at Berlin.—*Graefe and Walther's Journ. der Chir.*

8. *Preternatural Lactation.*—Aristotle gave immortality to the he-goat of Lemnos, when he affirmed, for the benefit of after-ages, that the generous animal yielded a profusion of milk, from which the goat-herd made excellent cheese. Very useful too, in this way, was "the wether which suckled a lamb;" and the history of this phenomenon is a contribution to philosophy, from the pen of Dr. Doddridge, alike distinguished for his sagacity and erudition, and piety.

Donati, in his "Marvellous History;" Paulini in his second "Century;" and the Bishop of Cork, related instances of the lacteous secretion being abundant in men.

Cardani enumerates the circumstance of a young woman, who had never been impregnated, whose breasts yielded milk, on their being excited with the stings of nettles. Dr. Stach, and Dr. Montegre have detailed cases of copious lactation accidentally re-produced in *aged* women: to these the present writer will add some account of a woman who gave suck to children, from the *twenty-fifth* uninterruptedly to the *seventy-second* year of her age: this person is still alive and accessible to examination.

1. *Dr. Stack's History.* Elizabeth Brain was examined by Dr. S. and another gentleman, at her house in Tottenham-court, in 1733: she was then in the 68th year of her age; and for more than twenty years she had not given birth to a child. Her breasts were "full, fair, and void of wrinkles; but her face and mouth was very much withered, her cheeks and mouth vastly sunk in, her eyes red and running with a clammy humor." Upon pressing her right breast at the Doctor's desire, "she fairly squeezed out milk which gathered in small drops at three of the lactaferous ducts terminating at the nipple." Having himself carefully dried the nipple with his handkerchief, Dr. S. made her repeat the experiment, and it had the same result.

About four years previous to this examination, E. B's daughter being obliged to be absent for a considerable time, left a babe she was then suckling, in the care of its grand-mother: the old woman, finding the child froward for want of the breast, applied it to her own, barely in order to quiet the infant, without the least thoughts of milk." This having been repeated several times, a son of E. B's, now grown a man, perceiving that the young one swallowed something from the nipple, "begged leave of his mother to try if he could not milk: his experiment succeeded, and he drew milk out of the same breast from which he had been weaned above twenty years; for seventeen or eighteen years previously his mother had not given suck to any child. Two years afterwards, E. B's daughter had another babe; whereupon its grandmother weaned the first, and commenced suckling the new-born infant. "In my presence, says the Doctor, this infant took the nipple with as much eagerness and seeming delight, as I ever perceived in a child of two years old; and at it plainly performed the actions of suction and deglutition: the two children, both girls, are, as to constitution, such as I could wish to the dearest friend; plump, and firm in flesh; in complexion cleanly, fair and healthy; and in temper brisk and sprightly, considering the mean diet of their nurse.
When this good woman came to town," the Doctor adds, "which was near two years since, her milk abounded to that degree in both breasts, that, to convince the unbelieving, she would frequently spout it above a yard from her: a particular which, among others, the good man and woman of the house, and others of the neighborhood, likewise, assured me of. Now her left breast is run dry, and she has no great quantity in her right; but what there is, is as good milk as one may desire in a nurse. The poor woman seems perfectly honest and artless, and even inclines strongly to dotage: she very religiously throws the whole upon a miracle."

*Dr. Montegre's History.* Having collected a number of analagous facts, Dr. M. published them in the 'Gazette de Sante,' in connexion with the following, which came under his own observation. "La Femme Charles," a delicate female, had male twins in 1810, but from the great weakness of her constitution, she could hardly supply one of them with milk; and in consequence of extreme poverty was unable to procure the services of a nurse. Affected by this discouraging embarrassment, says Dr. M., her mother, took one of the infants and put it to her own breast, which the babe instantly seized and began to suck. This ingenious grandmother was then in the sixty-fifth year of her age, and had been twenty-nine years a widow. At first her breasts yielded a fluid, in small quantity; but in a few days, her infantile grand-child drew from them an abundance of healthy milk. This tender nursling continued for two and twenty months to be nourished at its grand-mother's breasts; and throughout all that period was more vigorous than its brother who imbibed his sustenance from their common parent.

*Dr. K's History.* Judith Waterford lives in the village of Thringston, on the Forest, between Ashby-de-la-Zouch and Loughborough, Leicestershire. At this time, (*December*, 1831), she is in her eighty-first year, and though infirm, her constitution has singularly retarded the advances of old age. Through life, this woman has been the active and laborious inmate of a peasant's cottage; her person is short and well proportioned: at one time she weighed about 14 stone: her temperament is the neuro-sanguineous, distinctly marked. The peculiar circumstances of her history being extensively known in the district where she resides, she has been visited by many clerical, medical, and other curious inquirers.

J. W. had two husbands. Previously to her first marriage, her menstrual secretion appeared at regular periods; it continued nine days usually, and was very profuse, but preceded and accompanied with little pain or disturbance of the system. After this event, it returned only nine times, at distant and irregular intervals; and on each of these occasions it was abundant, without causing any perceptible diminution of her milk.

J. W. was first married in 1777; her first child was born in May, 1778; and from that period till May, 1825, her lacteous secretion never in the least subsided. Besides giving her breast freely and frequently to the young ones of her neighbors, she suckled six children of her own by her first husband, and eight nurslings: She had two miscarriages. Her first confinement was followed by an excessive uterine discharge, which continued three weeks and induced considerable debility. No infant however vigorous, was ever nearly able to use her supply of milk; and, in consequence, she often had her "breasts drawn" to the amount of two quarts in a day: her belief is, that, in general, she could have suckled four lively children at the same time. Many attempts were made, even under medical direction to suspend the secretion of her milk; but they all utterly failed. Her breasts often became tense and painful; and, for the removal of this state, she had them well rubbed with butter made from the cream of her own milk; this process was invariably advantageous. Sometimes about a pint of this cream was collected, and the butter it yielded was white, and soft as lard, with a sweet taste. Judy took much food, and seldom had recourse to medicine; it is her boast, indeed, that "she never paid a shilling in her life for doctoring for the sake of her health;" her bowels generally were torpid, often constipated.

J. W. was a widow nearly three years, and gave suck to her own and other children during the whole of that period. She had one still-born child to her second husband. Her breasts even now, retain a size quite extraordinary, and the axillary glands are occasionally large. Soon after the disappearance of her milk, in 1825, her health became more variable, her energies gradually failed, and her voice, which still is strong, lost its natural strength. Her actual powers of mind seem to have experienced little change; she continues "heart-whole;" her appetite is considerably impaired; she wishes to eat often, but takes little food at a time; her sleep is disturbed and unrefreshing, and a cough gives her occasional annoyance: her pulse is full, soft, not quick and resistant. For many months she has been slightly troubled with those periodical disturbances by which menstruation is preceded.

Although Judy's milk ceased, for a time, in 1825, it repeatedly appeared in minute quantities during the five subsequent years; but since the autumn of 1830, the secretion of this fluid has been constant, and, if encouraged, (she thinks), would be sufficient to nourish a child; from attempting this, however, she is deterred by the idea, that "it would soon be the end of her."

December 6, 1831. This day, being in the middle of her 81st year, Judy readily filled a small spoon with her milk, by squeezing her left breast frequently with the hand. This milk was rich and sweet, and not different from that yielded by young and healthy mothers.

Here, then are the remarkable circumstances of a woman who menstruated during lactation—who suckled children uninterruptedly through the full course of forty-seven years—and who, in her eighty-first year, has a regular secretion of milk.—*Medico-Chirurg. Rev.*

9. *Efforts of Nature to establish a new Joint after dislocation. Hip Joint. Dislocation into the Foramen Ovale.—Dissection.*—There is an excellent preparation of this accident in the collection at St Thomas' Hospital, which I dissected many years ago. The head of the thigh-bone was found resting on the foramen ovale, but the obturator externus muscle was completely absorbed, as well as the ligament naturally occupying the foramen, now entirely filled with bone. Around the foramen ovale bony matter was deposited so as to form a deep cup, in which the head of the thigh-bone was inclosed, but in such a manner as to allow of considerable motion; the cup thus formed, surrounded the neck of the thigh-bone without touching it, and so inclosed its head, that it could not be removed from its new socket without breaking its edges. The inner side of this new cup was extremely smooth, not having the least ossific projection at any part to impede the motion of the head of the bone, which was only restrained by the muscles from extensive movement. The original acetabulum was half filled by bone, so that it could not have received the ball of the thigh-bone if an attempt had been made to return it to its natural situation. The head of the thigh-bone was very little altered; its articular cartilage still remained; the ligamentum teres was entirely broken, and the capsular ligament was partially torn through; the pectinalis muscle and adductor brevis had been lacerated, but were united by tendon; the psoas muscle and iliacus internus, the glutei and pyriformis, were all upon the stretch. Nothing can be more curious, or, to the surgeon and physiologist, more beautiful, than the changes produced by this neglected accident, which exemplify the resources of nature in producing restoration.

*Dislocation backward, into the Ischiatic Notch.—Dissection.*—There is a good specimen of this accident in the collection at St. Thomas's Hospital, which I met wlth accidentally, in a subject brought for dissection. The original acetabulum is entirely filled with a ligamentous substance, so that the head of the bone could not have been returned into it. The capsular ligament is torn from its connexion with the acetabulum, at its anterior and posterior junction, but not at its superior and inferior. The ligamentum teres is broken, and an inch of it still adheres to the head of the bone. The head of the bone rests behind the acetabulum on the pyriformis muscle, at the edge of the notch, above the sacro-sciatic ligaments. The muscle on which it rests is diminished, but there has been no attempt made to form a new bony socket for the head of the os femoris. Around the head of the thigh-bone a new capsular ligament is formed; it does not adhere to the articulatory cartilage of the ball of the bone which it surrounds, but could, when opened, be turned back to the neck of the thigh-bone, so as to leave its head completely exposed. Within this new capsular ligament, which is formed of the surrounding cellular membrane, the broken ligamentum teres is found. The trochanter major is placed rather behind the acetabulum, but inclined towards it relatively to the head of the bone.

In this specimen, from the appearance of the parts, the dislocation must have existed many years; the adhesions were too strong to have admitted of any reduction, and if reduced, the bone could not have remained in its original socket.

*Dislocation on the Pubes.—Dissection.*—I dissected one of these dislocations, and we have it preserved in our anatomical collection. It shows changes of parts nearly equal to those of the dislocation into the foramen ovale. The original acetabulum is partly filled by bone, and partly occupied by the trochanter major, and both are much altered in their form. The capsular ligament is extensively lacerated, and the ligamentum teres is broken. The head of the thigh-bone had torn up Poupart's ligament, so as to penetrate between it and the pubes. The head and neck of the bone were thrown into a position under the iliacus internus and psoas muscles; the tendons of which, in passing to their insertions over the neck of the bone, were elevated by it, and put on the stretch. The crural nerve passed on the fore part of the neck of the bone upon the iliacus internus and psoas muscles. The head and neck of the thigh-bone were flattened, and much changed in their form. Upon the pubes a new acetabulum is formed for the neck of the thigh-bone, the head of the bone being above the level of the pubes. The new acetabulum extends upon each side of the neck of the bone, so as to lock it laterally upon the pubes. Poupart's ligament confines it on the fore part; on the inner side of the neck of the bone passed the artery and vein, so that the head of the bone was seated between the crural sheath and the anterior and inferior spinous process of the ilium.

*Shoulder Joint; dislocation in the Axilla.—Dissection of an old Dislocation.*—The head of the bone is found altered in its form; the surface towards the scapula being flattened, a complete capsular ligament covers the head of the os humeri. The glenoid cavity is completely fiilled by ligamentous matter, infused by a slow inflammatory process; in this ligamentous matter are suspended small portions of bone, which appear to be of new formation, as no portion of the scapula or humerus is broken; a new cavity is formed for the head of the os humeri on the inferior costa of the scapula, but this is glenoid, like that from which the os humeri had escaped.

*Dislocation forwards.—Os Humeri; appearances on dissection.*—The head of the os humeri is, in this accident, thrown on the inner side of the neck of the scapula, between it and the second and third ribs. I have had no opportunity of dissecting a recent accident of this kind; but in the Museum at St. Thomas's Hospital there is a beautiful specimen of one in a limb which had been long dislocated, and which was removed from the shoulder of a patient by Mr. Green, and dissected by Mr. Key, who gave me the following account of the appearances:—The head of the bone was thrown on the neck and part of the venter of the scapula, near the edge of the glenoid cavity, and immediately under the notch of the superior costa; nothing intervened between the head of the humerus and scapula, the subscapularis being partly raised from its attachment to the venter. The head was situated on the inner side of the coracoid process, and immediately under the edge of the clavicle, without having the slightest connexion with the ribs; indeed, this must have been prevented, by the situation of the subscapularis and serratus magnus muscles between the thorax and humerus. The tendons of all the muscles attached to the tubercles of the humerus were perfect, and are shown in the preparation. The tendon of the biceps was not torn; and it adhered to the capsular ligament. The glenoid cavity was completely filled up by ligamentous structure; still, however, preserving its general form and character. The tendons of the supra and infra spinatus, and teres minor muscles, adhered by means of bands to the ligamentous occupying the glenoid cavity: and to prevent the effects of friction between the tendons and the glenoid cavity in the motions of the arm, a sesamoid bone had been formed in the substance of the tendons. The newly formed socket reached from the edge of the glenoid cavity to about one third across the venter. A complete lip was formed around the new cavity, and the surface was irregularly covered with cartilage. The head had undergone considerable change of form, the cartilage being in many places absorbed. A complete new capsular ligament had been formed.

The pectoralis minor is not mentioned in this dissection; but from the natural situation of the coracoid process, into which this muscle is inserted, it must have passed over the head of the os humeri, as did the pectoralis major.—*Cooper on Dislocations.*

10. The following quotation, from Godman's Anatomical Investigations, will go far towards illustrating the principle of the formation of a new capsular ligament around joints in unreduced dislocations.

*On the formation of the Capsular Ligaments of the Joints.*—Anatomists hitherto have uniformly considered the *capsular* and *proper ligaments* of the joints as similar in character, and, like the latter, being merely continued from a point of origin on the extremity of one bone to an insertion near the extremity of another. By the *proper* ligaments I mean those that are solid, fibrous, and mostly perpendicular, resembling the lateral ligaments of the knee joint and those from the fibula and tibia to the tarsal bones.

The capsular ligaments, however, must be separated from the proper ligaments, inasmuch as they are immediately related to the muscular system, being formed by the layers of the fasciæ which sheathe all the muscles, or fairly encircle them.

The muscles of the upper part of the body and all the trunk, not excepting the heart, are contained within duplicatures of the fascia superficialis. The same fascia forms the *capsular* ligament of the shoulder joint, by the succession of the layers forming the sheaths for all the muscles about the scapula going to be laid out over the joint, and by their aggregation constituting the thickness of this capsule.

The fascia lata femoris sheathes all the muscles of the inferior extremities, and the duplicatures, enclosing the muscles of the thigh, form the great capsule of the hip joint, as minutely described in my Anatomical Investigations recently published. On the same principle the capsular ligaments throughout the body appear to be formed from the fasciæ sheathing the muscles. For the details I must refer to the work above named, and the 18th No. of the Philapelphia Medical and Physical Journal, in which the details of the formation of the capsular ligament of the shoulder joint will be given.

The muscles within the belly and pelvis derive their sheaths from the *fascia interna abdominis*, heretofore called *fascia transversalis*, which also forms the ligaments of the liver and bladder, the prostrate fascia, &c.

Some persons in speaking of the *fascia superficialis* state that it is composed of condensed cellular membrane. This is correct as applied to its ultimate analysis, but not when we examine it in the recent subject. It is equally true that the skin, tendon and bone, are composed of cellular texture, but we only arrive at this conclusion by a long and careful series of experiments.

The discovery of the formation of the capsular ligaments by a thorough *analsyis* of the fasciæ, leads to a variety of important conclusions relative to their physiology and pathology, and gives us a better idea of their relation to the strength of the muscles, whose actions are all modified by the manner in which they are sheathed by these fasciæ. See 18th No. of Philadelphia Medical Journal.

11. *Singular case of Lacerated Wound.*—By James Deane, M. D., of Greenfield, Massachusetts.—Mr. J., about twelve weeks since, while standing near the end of the arbor of a heavy grindstone revolving rapidly by water power, had his clothes caught by it and twisted up like a thread upon the spindle. This twisting commenced near the genital organs, and immediately involved them in the operation, in such a manner as to remove every portion of integument and cellular membrane from the penis and upon the pubis, in a circular line from one external ring to the other. Every particle of the scrotum was torn away, leaving the cremaster muscles and tunicæ vaginales completely naked. The right spermatic cord was affected with involuntary twitchings; but the left appeared considerably elongated, and to have sustained considerable injury. Both cords were entirely detached from all connections up to the external rings from which they hung, and a better display of the parts, or clearer dissection, could not have been made by the most expert anatomist. The sensibility of the denuded parts was most intolerable; the gentlest touch produced exquisite suffering. The circumference of the injury was well defined resembling more an incised than lacerated wound; it was regular in its outline, extending from two inches above the penis to the perineum, and in depth to the external fascia. A penknife with three blades, which went into the operation, was bent into a circle and the blades broken, showing the great power which well nigh spun his thread of existence to an end. Had not the machine been soon stopped, the penis and both testicles must inevitably have been torn away; as it was, it was a frightful injury, and in its nature, perhaps, without a parallel.

In this state of things the medical gentlemen of the vicinity were immediately assembled, and from the singularity of the case some embarrassment was experienced in deciding upon the best course to adopt. It was, however, decided that as the left spermatic cord had sustained severe injury, and as its loss would not necessarily cause emasculation, it should be removed. In this trying situation the fortitude of the patient did not forsake him; he entreated that this measure should be adopted, if his safety in any way depended upon it. The operation was easily performed by holding the cord firmly between the thumb and finger and dividing it with a bistoury, and tying a single artery which bled very freely. Upon relaxing the pressure, the spermatic vessels were felt to recede through the fingers; and, as a precautionary measure, a ligature was loosely applied to the remnant of cord to prevent its recession into the abdomen, which, however, did not take place. The wound was then dressed by wrapping the parts in the lightest linen wrung from a liniment composed of equal parts of lime water and of olive oil. This dressing was found exceedingly useful in protecting the denuded surface from irritation, without evaporating. The penis and remaining testicle were suspended in bandages passing over the legs.

Nothing could exceed the sensibility of this wound. The slightest motion, or touch, or drop of urine, would excite intolerable suffering. The treatment was completed by observing the strictest antiphlogistic rules. The cure went on well enough, but was a serious business for the patient. The moment he went to sleep, he was troubled with erection, which caused excruciating pain. This was not all; an induration of the inferior surface of the penis added chordee to this symptom, and these continued for more than two months. Doubtless this erection was a desirable circumstance for obvious reasons; still it was most tormenting, causing bleedings and short and disturbed sleep.

Although the progress of the cure was slow, yet it was wonderful to witness the resources and operations of nature. The testicle, which swung from the external ring, united to the surface of the wound in the perineum; the cavity of this wound was filled by granulations, and covered in part by the contraction of the skin from its edges, and partly skin newly organised, as was the penis (but without prepuce) and anterior surface of the testicle. The testicle is well provided for, being buried and protected in the perineum; the integument now covering the penis is loose and ample, which is to be attributed to the erection, for two months such a source of torment.

Mr. J. is a young man in robust health, and submitted with great resolution to all the painful emergencies in which this unfortunate accident placed him; and I have no doubt but he will ultimately be restored to his full procreative powers. This case, too, gives a useful lesson upon the restorative powers of nature; and it appears to me that we do not place reliance enough upon this wonderful provision, and that we too often lay down science to take up the knife.—*Boston Med. and Surg. Jour.*

12. *On the Sleep of Plants.*.—M. Virey, in a memoir entitled *Flore Nocturne*, (Flora Nocturna,) announces the following results or laws, which he has deduced from his researches on this subject. Cold and humidity diminish the transpiration of vegetables; the sap, then, instead of ascending to the summits of the leaves and flowers, as during the day, descends towards the roots. Hence, the sap vessels of those parts, frail and fine as they are in many plants, become almost empty and contract by their own elastic force. This is the reason why so many compound flowers, the Malvaceæ, the Convolvuli, &c. close during the night, or even when the sky is covered with clouds. For a similar reason, a numerous class of plants with pinnated leaves, fold them and sleep during the night. The returning warmth of the sun again sets the sap in motion, and again invigorates the leaves and petals. The heat and light dilate the vessels with a sort of turgescence, and expand the foliage until the return of night again drives the sap from their delicate vessels. But why is it otherwise with nocturnal plants, which appear to languish and to be overcome during the day, and unfold their beauties only when the sun is withdrawn? It is because his ardor acts too powerfully upon the frail texture of certain petals—evaporates too rapidly their nutricious juices, and causes them to close. But during the freshness of the night, these juices remain in the tissue of the plant, fill their tubes, and unfold their surfaces to the atmosphere. *Rev. Encyc.*

13. *Ilicine—a Remedy in Intermittent Fever.*—Doctor Emile Rousseau has just published his own observations, together with those of eminent practitioners in civil and marine hospitals, as well as those of various private physicians no less estimable, all uniting in ascribing to the leaves of the common Holly (Ilex aquifolium) great efficacy in the treatment of intermittents. They consider this indigenous plant as the powerful succedaneum of quinquina and the sulphate of quinine. Several of them agree in considering the holly as superior to quinquina. Dr. Rousseau deserves great credit in bringing the virtues of this plant so fully into notice. He has succeeded in obtaining its active principle in an isolated form, and has given it the name of *Ilicine.—Rev. Encyc.*

14. *Regeneration of Nerves.—Remarks on the Regeneration of Nerves.* By F. TIEDEMANN.—Divided nerves unite and heal. This fact has been established by the experiments of Fontana, Michaelis, Arnemann, Cruickshank, Haighton, Mayer, by Bychat, and by the more recent observations of Swan, Descot, and Larrey.

The re-union of divided nerves is attended by the following phenomena:—the ends of the nerve retract; the extent of the separation is from two to six lines or more, and is greater in large nerves than small ones. This separation of the extremities of a divided nerve is not owing to elasticity, but is the result of an organic action, or contractility of the neurilema and surrounding cellular tissue; in proof of which it may be stated that divided nerves in a dead animal do not retract. Inflammation soon commences; the surrounding vessels contain more blood; the nerves become of a red color, and are thicker; the inflammation extends from half an inch to an inch above and below the divided extremities: the distension of the vessels, the redness and swelling, are, however, more remarkable in the upper than the lower end. Coagulable lymph is deposited around the separated nerves, and in this lymph minute vessels are observable. In consequence of this deposition in the sheath of the nerves, and among their fibrils, the nerves appear enlarged, the swelling is greater in the upper than in the lower ends. Similar enlargements are discoverable after the lapse of time, in the ends of nerves divided in amputation.

The coagulable lymph effused during the inflammation, connects the divided nerves in the course of a few days; it gradually assumes a further consistence, and the blood-vessels dispersed through it appear to contract, or to contain less blood. The enlarged or bulbous extremities of the nerves, gradually approaching nearer to each other, at length become incorporated, and thus the connection of the divided nerves is re-established. If the swelling be examined after some time, it is found reddish externally, and exhibiting fibrils similar in appearance to the nervous fasciculi, and by means of these the nerves become perfectly continuous.

Whether the substance connecting the nerves is similar in organization to the original nerve, and is capable of transmitting sensation, and the influence of the brain in the performance of voluntary motion, are questions which have divided the opinions of physiologists. Arnemann rejected the opinion that the regeneration of true nervous substance took place, having found, that 150 days after their division, the parts supplied by such nerves, were destitute of sensation. Breschet, Richerand, and Delpech adopted the same view.

Fontana, on the contrary, Michaelis, Mayer, Cruikshank, and Haighton, maintain that the re-union of divided nerves take place by means of true nervous fibrils. Michaelis recognized these fibrils by the microscope, and Mayer demonstrated them by the test of nitric acid. Haighton, in proof of the regeneration of nervous matter, stated that the re-united *nervi vagi* were capable of performing their natural functions. He divided the nervus vagus of the one side in a dog, and in six weeks afterwards, that of the other side. The animal lived; but when the two nervi vagi were divided at the same time, or in a shorter interval, the animals invariably died.

From the preceding experiments and observations, Tiedemann considered it highly probable, that true nervous matter was regenerated; he thought, however, that the return of sensation, and the power of motion in parts whose nerves had been divided, was not established so satisfactorily as was desirable. He therefore instituted some experiments on the subject, and one of the most conclusive of these he has detailed nearly as follows. On the 16th of August, 1827, having exposed the brachial plexus of nerves (*arm-nerven-Geflecht*) in a dog, he separated the several nerves, and cut out of each a portion from ten to twelve lines in length. The leg and foot were immediately deprived of sensation, and the power of motion. The wound healed within three weeks, but the leg and foot remained without sensation, or the power of motion, for a long time. It became smaller than the opposite one, and, in walking or running, was drawn upwards by means of the muscles of the shoulder.

In May, 1828, eight months after the excisions of the portions of nerves, the animal began to use the foot in progression, and showed signs of sensation when it was much pressed, or was pricked with needles; and during this and the following years, sensation and motion were gradually but perfectly restored. In order to examine the condition of the nerves, the animal was killed on June 2d, 1829, twenty-one months after the operation. Where the portions of nerves had been removed, at each extremity of the incision an oval enlargement was found, which was greater at the end nearest the body than the opposite one. In the interval between these enlargements, and connecting them, newly formed portions, apparently of nerve, were seen. These intermediate portions were thinner than the uninjured parts of the nerve. In order to ascertain whether the regenerated parts really consisted of nervous fibrils, a portion was laid on a piece of glass and nitric acid applied, but the integrity of the structure remained unimpaired. Hence, from the return of the power of motion and sensation, from the structure of the newly-formed portion, and from the test of nitric acid, Tiedemann concludes this experiment to supply a demonstration of the regeneration of true nervous matter.

Numerous cases are related, for the most part by English authors, (as Abernethy, Balfour, Pring, and Swan,) of the restoration of sensation after the healing of wounds, in which nerves were divided either by accident, or in operations for the relief of neuralgia. Of these the most remarkable is related by Abernethy; and one very analogous to it, has been communicated to Tiedemann by Dr. Shott, of Frankfort.

A woman, 40 years of age, had suffered most severely for fourteen years, from neuralgia of the ring-finger, particularly of the last joint, for which she could not obtain any relief. Dr. Shott determined, therefore, to remove a portion of the cubital nerve. He laid bare the trunk of this nerve above the inner condyle of the humerus, and removed a portion of it an inch long. After the division of the nerve, the neuralgia immediately ceased, and the ring and little finger of that hand were deprived of sensation. In order to prevent the re-union of the ends of the nerve, the wound was dressed to the bottom, and healed by suppuration. After three months the wound was cicatrized, and there was no return of neuralgia; but gradually sensation returned in the fourth and fifth fingers, and after six months had elapsed, she again suffered severe pain in the ring-finger, which, however, did not become so violent as before the operation.—*Lond. Med. Gaz.*

15. *The Diving Bell a Remedy for certain Diseases.*—An interesting account of the descent in a diving Bell of Mr. Clifford, of Exeter, N. H. and Capt. Tripe, of Dover, is published in the last number of Silliman's Journal, in which the Reverend author states some facts leading him to the conclusion, that excursions of this kind may be useful in cases of Rheumatism. The pulse, on emerging from the bell, was quick, and perspiration very profuse, and the gentlemen found themselves in a fit condition for a comfortable sleep. It seems, therefore, not unreasonable to expect some relief in cases requiring stimulating diaphoretics, and to which quiet repose is so greatly desired; but since the common sulphur bath accomplishes these objects in a much more speedy and convenient manner, we can hardly expect that the diving bell will be a very general resort for these purposes. The fact, however, of the peculiar influence of these descents on the several functions, should be known to medical men; and we will close this part of our notice of it, by a brief extract from the interesting paper of Mr. Alden.

Mr. Clifford had, for many years, been afflicted with rheumatic pains. During the several weeks he was engaged in this enterprise, *he was remarkably free from this complaint.* The first time he descended in the diving bell, he happened to be considerably affected with his disorder; but, on coming out of it, he was entirely relieved from pain, insomuch that he walked, directly after, six miles, without inconvenience. This was an exertion which he had not thought himself able to make for several years before.

Could a series of experiments be instituted, on proper subjects, who will venture to say that the result would not be so much as to render a sub-marine descent, in a commodious diving bell, a frequent and favorite adventure?

But this is not the only view in which these excursions are to be considered by the physician. The very singular effect they have long been known to produce on the organ of hearing, may possibly lead to more useful results.

In descending, says Mr. A. a painful sensation was induced on the tympanum, attended with a noise, as Mr. Clifford informed me, not unlike that of a fly entangled in a spider's webb, till the adventurers were at the depth of about twelve feet, when, experiencing a sudden shock, they were completely relieved. This painful sensation, the shock, and subsequent relief, were regularly repeated, as nearly as could be judged, every twelve feet. After a few descents, it was perceived that, by being raised a foot or two, every eight or ten feet, the shock was avoided, and the men were freed from that painful sensation, which had resulted from the uniformly increasing density of their atmosphere.

Upon the above, Dr. Mease, who communicated the account to the American Journal, makes the following remarks:—

The painful sensation in the ear, mentioned in the preceding paper, is invariably experienced by those who descend in diving bells, owing to the compression of the condensed air on the membrana tympani; but the means of preventing it, which were discovered by Messrs. Clifford and Tripe, are not mentioned in any of the accounts of diving which I have read, nor do the writers of them notice the 'shock' felt by the Portsmouth divers which immediately preceded their relief from the pain. Dr. Hamel, of St. Petersburgh, states that he was relieved of the pain by making exertions to admit air through the Eustachian tube into the ears, but succeeded in accomplishing this at first only on one side, when the air rushed into the cavity of the right ear, and the pain ceased instantly; when in the diving bell, he was not aware of the simple way in which it was effected. Dr. Wollaston informed him, that nothing is wanted but to swallow the saliva, as may be seen from the following simple experiment. Close your nostrils with the fingers, and suck, with the mouth shut: air will come through the Eustachian tube from the ear, and you feel pressure on the membrana tympani, which prevents you from hearing distinctly. As the end of the tube nearest to the mouth acts like a valve, this sensation will often remain even after you have ceased sucking. To remove it, nothing is wanted but to swallow saliva, whereby the action of the muscles seems to open the end of the tube, and then the air rushes in to re-astablish the equilibrium. During the descent of the bell, Dr. Hamel says that the pain returned, but as he repeated his exertions to open the Eustachian tube, the air at intervals found a passage through it, and he obtained relief. Through the left Eustachian tube no air had yet passed, and the pain in the left ear was gradually increasing; when about fourteen feet under water, the sensation was as if a stick was forced into the ear from without: at last, during one of the exertions to open the mouth of the tube on that side, the air forced its way in with considerable violence through it, and he was relieved from the pain also on that side. I presume the 'shock' experienced by the Portsmouth divers, arose from the rushing of the air into and through the tubes, as it took place immediately preceding their obtaining relief from the pain in their ears. It may be useful to state, that this pain will be much diminished, if the bell be allowed to descend slowly, so as to admit the air gradually into the ear. In ascending, Dr. Hamel says that the pain returned, resulting from the air of the inner cavity of the ear expanding, as the external pressure was diminished; but it was more easily relieved, the air gushing occasionally from the ear through the Eustachian tubes into the mouth.

Dr. H. suggests the probability of the diving bell being used with success for the cure of deafness, in those cases where it depends on an obstruction of the Eustachian tube. The patient would have to go down in a diving bell, and make exertions to open the mouths of the Eustachian tubes, and then by the pressure of the condensed air it would be forced through the extent of the tube, and thus clear the passage. He thinks that the fact of such slight obstructions having been frequently removed by forcing air or tobacco smoke from the mouth into the ear, gives weight to the idea: but it is questionable whether the deaf person would be able to bear the great pain which it is reasonable to suppose he must endure from the condensation of the air on the tympanum, until the removal of the obstruction; and his sufferings might be so great as to deprive him temporarily of his presence of mind, and even of his senses. The experiment ought not therefore to be made, unless another person enjoying his hearing accompanied the patient. I have more well grounded confidence in syringing the ears with warm milk and water, to remove hardened wax. The relief experienced by Mr. Clifford from the rheumatism, after his diving, is well worth consideration by the faculty.—*Bost. Med. and Surg. Jour.*

16. *Observations on Club Feet.*—M. Dupuytren, lately exhibited at his clinical lecture an infant born in the Hotel Dieu, having two strongly marked club-feet. The learned surgeon took the opportunity of making the observations which follow.

Club-foot is for the most part a congenital deformity, in which the foot is turned very much inwards, the sole being nearly perpendicular, and the external edge looking down, so that the individual rests upon it, or, when the deformity is very great, even on the outer ankle. At the same time the cavity of the sole of the foot appears to be augmented. All these external phenomena have been well described by Scarpa and others. Scarpa and M. Cruveilheir have also occupied themselves with the interior appearances displayed by dissection, but none of them have sufficiently dwelt on the most important results of the club-foot—namely, the diminished nourishment and atrophy of the limb.

Congenital club foot is either limited to one or involves both members. In the former case, if the infant be examined very soon after its birth, the deformed foot is found to be rather smaller than the other, but the legs are of equal length. M. Dupuytren has had numerous opportunities of satisfying himself on this point. When both feet are affected, they are in general equal in point of development. This kind of premature atrophy, the unknown cause of which is probably connected with that of the deformity itself, produces as a consequence a secondary wasting, which extends to the entire limb, and the source of which may be better explained. In fact, the infant, from the day he begins to walk, constantly makes use of the sound limb chiefly, resting the weight of the body almost always upon it. The nutrition bears proportion to the exercise; while the other limb, almost unemployed, wastes, in consequence of its inactivity.

Now this atrophy takes place in two different ways, which have hitherto been confounded, but which ought to be distinguished. 1. The limb wastes in length; 2. it wastes in thickness. The wasting in breadth is but little manifested in the skeleton, though much in the muscles, and hence the weakness and thinness of the limb—a vexatious circumstance, it is true, but one which may be remedied by calling the parts into exercise. The atrophy, as to length, takes place both in the bones and in the muscles, but is developed in the skeleton, which is most important, and thence proceeds a shortening of the limb, which no remedy can cure. The difference in length between the limbs increases in direct proportion to the age. Not perceptible at birth, it becomes obvious some years after; and at the age of ten, M. Dupuytren has always found it well marked, and still more so at twenty, if no means of prevention have been adopted.

The shortening of the muscles and tendons, less considerable in general, becomes nevertheless irremediable after a certain time. Thus at twenty, the tendo-achillis has so lost its due length, that when the foot has been brought to its natural direction, the heel continues to be pulled upwards, so as to oblige the individual to have a very thick sole to the heel of his shoe, in order to be able to rest at all upon the part. It is therefore desirable to prevent as much as possible this shortening of the bones, because when it once takes place it is irremediable. According to these views, M. Dupuytren requested several practitioners who devote themselves especially to such cases, to begin the treatment immediately after birth: he sent four or five infants of very early age, to an establishment for 'Orthopedy,' where they remained not more than five or six weeks. The deformity was completely corrected, so that the children learned to walk in the usual manner; and the limb, never having lost its length, performed its functions in the usual manner. I witnessed the results, said M. Dupuytren—I kept the individuals in view—and ascertained that the deformity did not return; and I affirm that, by treating club-foot thus early, the wasting of the limb may be avoided with certainty.

It may be added, that the younger the infant is, the more readily will the foot yield to pressure. In a new-born babe the hand of the surgeon is sufficient to restore its natural form to the foot, and that without causing any pain. A few months more, however, increases the difficulty; and when the patient has passed his tenth year, machinery is required to accomplish the end in view. After twenty, even mechanical contrivances fail to be of service. This depends upon three principal circumstances: 1. The suppleness of the ligaments and muscles, which continues to diminish as the age increases; 2. The increase of the difficulty itself; 3. The imperfect formation in which the bones are developed, which last is decidedly the most powerful cause.

In conclusion it may be stated, that the treatment of club-foot, if undertaken at birth, is easy and simple: it is both completely preventive and curative.—*Bost. Med. and Surg. Jour.*

17. *The Resurrectionists.*—It has been our belief that no possible method could be devised for rendering secure the sacred mansions of the dead, but that which was recently adopted by the Legislature of Massachusetts, and soon after by the Parliament of Great Britain. In both countries the remedy has been effectual. By abolishing the office of the resurrectionist, it rids the community of the whole band of speculators, from whom alone violation of the grave could be apprehended; the remains of mortality that rest in the earth, repose there in security, and the minds of surviving friends are no longer harassed by the fears that, in times past, added so much to the poignancy of grief at the death of a relative or friend. Such fears were well grounded; they were rational, and it is therefore well that they are hushed. No man who possesses any thing of a philosophical mind, would object that his body, after death, should subserve the purposes of science and humanity; but it is quite a different thing to contemplate one's mortal part deposited as a temptation to crime, and made an article of speculation. The apprehensions, therefore, that grew out of the former state of the law, respecting dissection, are, in more than one view of the subject, entirely put at rest. The grave is effectually protected, and the means of anatomical knowledge supplied, and the only circumstance attending dissection that could disturb the dying or annoy his friends, necessarily dispensed with.

The following notice in an English Journal illustrates the effect of the late law on this subject, in England.

Some of those desperate men who have heretofore lived by the plunder of the grave, have been endeavoring, in various ways, to annoy and disturb the anatomical teachers in the metropolis. In some instances, they have endeavored to create disturbance, with a view of extorting money; in others, they have tried to obtain employment at their old occupation, offering to furnish 'subjects' for two pounds each, instead of twelve, the average price of last year. Thinking the temptation would be too strong to be resisted, a party of them last week, went one night to several schools with two bodies, offering them for anything or nothing: at last they pressed one gentleman for heaven's sake, to take the bodies, were it only to bury them, for they were fearful of being detected, as no one would give them admission. We can confidently state, that the determination peremptorily to decline all connection with these men, has not been deviated from in a single instance, and thus the public has been the first to gain by the new act; for the temptation to rob the grave ceases with the possibility of disposing of the plunder, and thus may the friends of the deceased feel assured that their '*requiescat in pace*' will not be in vain—that hereafter, the grave will, indeed, be sacred.—*ib.*

18. *Effects of Darkness in producing Deformities.*—A correspondent writes us the curious fact. There is at present in Paris an artist of the Louvre, an eminent historical painter, of the name of Ducornet, who paints with his feet. He was born without arms, of poor parents, at Lille. There are also about the French metropolis a number of beggars, twelve or thirteen of them at least, all deformed in various ways, and all born at Lille, in certain dark caverns under the fortifications. The effect of these places, from their want of light producing malformed births, is so notorious, that the magistrates of Lille have issued strict orders to prohibit the poor from taking up their abode in them. It is added by our correspondent, that he had a conversation with Mr. Edwards on the subject, and that gentleman was greatly struck with the confirmation which the above circumstances afford to his views, stated in his work, *Sur l'influence des agens physiques sur la vie.* Mr. Edward's experiments of detaining tadpoles in darkness and thus causing them to grow into gigantic and monstrous *tadpoles*, instead of being transformed into frogs, are well known.—*London Medical Gazette.*

19. *Insomnolence cured by Sulphate of Quina.*—Mr. Barbier, of the Hotel Dieu, Amiens, relates the following case:—A man, aged 42, had cholera, from which he recovered; all the functions were restored, except that his sleep was destroyed: scarcely had he an hour's rest altogether in the course of each night; laudanum and other soporifics were exhibited without effect. Mr. Barbier found, on examination, that every evening he had a nervous 'agitation,' which lasted all night, accompanied by some pain in the head and limbs. Looking to the *periodicity* of the affection, M. Barbier ordered six grains of sulphate of quina every night. It was given two nights; he slept well; it was then omitted; he had no rest. The medicine was again renewed, and continued, with the effect of permanently procuring six or seven hours of sound sleep.—*Gaz. Med.*

20. *Treatment of Scrofula by Iodine.*—M. Baudelocque, physician "de l'Hopital des Enfans," communicates an interesting memoir on the results of his practice in strumous disease. Sixty-seven girls, from the age of four to fifteen years, were treated by iodine in its different preparations. In all, the disease had existed for a considerable time, varying from nine years to ten months. Fifteen of these children were completely cured; fourteen so much improved, as to indicate a speedy cure: thirteen experienced less benefit than the preceding, but still sufficient to warrant hopes, though distant, of their recovery; five exhibited little, and twenty no amendment at all. All these patients with scarcely an exception, were much worse when they commenced the use of the iodine, than at the period of their admission into the hospital. Far from having derived any advantage from their residence there for several months, or even years, their condition was manifestly less favourable. This remark is well worthy our study, as it disproves the assertion of those medical men, who state that scrofula has a definite duration in the system, and that, after a certatn lapse of time, it is easily cured, whatever remedies are employed. M. Baudelocque is satisfied, that the length of time during which the disease has existed in any case, had no influence in assisting or in quickening the cure of the numerous patients under his care.

Of the 67 cases, 17 had an enlargement of the glands of the neck, jaw, and arm-pits; they took iodine inwardly, and employed likewise ioduretted baths, and friction with ointments of the potassæ hydriodas, plumbi ioduretum, et hyrargyri proto-ioduretum.

The mode in which the discussion of these enlargements is effected, makes us well acquainted with the nature of their formation; they consist of an aggregation of numerous small tumours, united together by cellular texture, which, in course of time, becomes firm, and increased in extent. In proportion as this cellular substance is dispersed, and returns to its normal state, the small glands, which are less quickly absorbed, become moveable and disjoined, and these, again, are resolved into several still smaller, until they quite disappear. To prevent the deformity of the scars so frequently left after a scrofulous abscess, the loose skin must be destroyed, either by the knife or by caustic, during the cicatrization of the sore, and a new healthy skin will be formed, so as to cause little disfigurement.—*Revue Medicale.*

21. *Natural History of Man.*—M. Geoffrey Saint Hilaire concludes an interesting memoir by stating that females are generally much shorter than the males, in those races of mankind whose stature is great; the difference being much less among the people of low stature; that the most remarkable for tallness, for the most part inhabit the southern hemisphere, while the most diminutive are found in the northern one; that among the very tall races some live in South America, others in the South Pacific Archipelago, between the 10th and 50th degree of south latitude; that some very diminutive races live in the southern hemisphere; and that some tall races are found in the northern hemisphere; and if we compare the geographical position of these various divisions of mankind, we arrive at the curious result, that the diminutive races are almost always found near the verv tall, and the very tall races near to those of low stature; that the variation in the size of different races may be explained in part by the influence of climate, diet, and mode of life—that it is extremely probable that the stature of the human race has not sensibly diminished, since the earliest age of history, or even from long anterior to that epoch.—*Ib.*

22. *Medical Jurisprudence. On the Decomposition of the Animal Structure.*—M. Oliver, and M. Denis were desired to examine the body of a female, who had been interred three months before, as suspicions had been excited that she had been murdered by her husband, and had died of the effects of blows upon her head, and fracture of the skull. The husband alleged that he had obtained the authority for interment from the physician appointed to ascertain the cause of his wife's decease; but it appeared that the physician had never examined the corpse, but had trusted to the husband's declaration that she had died from a rupture of a blood-vessel. The deceased was 55 years of age, and had been affected with hemiplegia for nine years. The body was buried the day after her death, in a dry chalky soil.

When exhumed, it did not present the appearance of much decay; the external surface, and the pectoral and abdominal viscera were little altered; but on examining the head, the right hemisphere was found softer and more decomposed than the left one, the anterior lobe was converted into a fatty matter, semi-consistent, and quite similar to what covered all the surface of the brain and spinal marrow, in the place of the pia mater, which had altogether disappeared. A similar greasy substance was discovered in both pleuræ, posterior to the lungs, which were quite shrivelled and dry. M.M. Oliver and Denis were led to the conclusion, that adipocire was formed at those parts where blood, or a bloody fluid had been deposited, and that, therefore, this female had died of a cerebral hæmorrhage. Such was their opinion, founded partly on the greater softness of the right hemisphere, and partly on the fatty degeneration of the anterior lobe, which indicated that it had been the seat of the hæmorrhage; else how was the fatty deposit not found in other portions of the cerebral substance? The occurrence of a similar matter in the two pleuræ is easily accounted for on the same view of the subject; for we know that in rapidly fatal apoplexies, there is generally an excessive congestion of blood in one or in both lungs; and it is but probable to suppose that after death there may be a gradual sudation of the same into the cavities of the pleuræ.

The husband was acquitted on the strength of the medical evidence. —*Ibid.*

23. *Rhinoplastic Operation.*—The patient had been long affected with an enormous cancer of the nose, which had resisted every treatment. M. Blandin, having completely excised the diseased part, detached from the forehead a flap of integument, and shaped it to the stump of the nose; but he did not divide the pedicle of the flap, as has been usually done; instead of doing this, he separated the skin from it, and then cut away the integuments from the root of the nose; the opposite raw surfaces were brought together, and quickly united. By this maneuvre, the new nose continued to retain its communication with the blood-vessels and nerves, which imparted life to it at first, and was thereby much stronger and less likely to be affected by cold and other accidents.—*Journal Hebdomad.*

24. *Curious Case, in which the real Sex of a Child was not discovered till he was* 18 *years old.*—E. D——c, was born on the 13th August, 1812, and appeared to be a female, was registered as such, and educated as a girl till 1831. In June of this year she consulted M. Gendrin on account of opthalmia in both eyes. He was struck with the beard on her face, the bass tone of her voice, and the development of the pomum Adami. The catamenia had not appeared. Subsequently she mentioned that there was an irregularity in the genital organs, and that the irregularity had been increasing ever since the age of puberty, which with her commenced about six years before, as indicated by the change of voice, and the black, stiff hair which appeared in different parts of the body. The mother stated that her child, when young, had the activity, and had always preferred the sports of boys, to the quieter amusements of girls.

She has not shewn any attachment for either sex. On examining the genital organs, a longitudinal fissure, like a valve, extended from the inferior edge of the symphisis pubis to near the anus. Rather higher up than the usual situation of the clitoris, was observed a loose cylindrical organ, 15 lines long and about five or six thick, covered by a very fine and extensible membrane, and ending in a regularly formed glans, having a corona round its base. The vena dorsalis penis was seen on its upper surface. A prepuce covered one half of its surface; the skin of which this prepuce is formed, was continuous with the margins of the vulva-like fissure in the perineum. Along the under surface of this organ was a deep channel, or groove, which terminated posteriorly in the vulva, and at this point of junction were observed five open, irregularly arranged orifices, not unlike those of the vasa efferentia round the verumontanum. A smooth mucous membrane covered the surface of this gutter, which was in lieu of a urethra, and it presented faint traces of the longitudinal furrows observed in the natural passage. With much difficulty the finger could be introduced to the depth of three inches, into the vulva, but no opening was detected in it. A straight instrument might be passed in the direction, from the symphisis pubis to the middle of the sacrum, for three inches and a half; and when the finger was carried up the rectum, the instrument might be felt, and it was ascertained that there was not more than two or three lines thickness of substance interposed. Two elastic roundish bodies were felt by the finger round the supposed neck of the bladder; these were probably the lobes of the prostate. No meaus urinarius could be perceived; but, as the water was discharged downwards, the orifice must have been situated in the perineal canal. At first, this canal was considered to be merely a dilated urethra, but the circumstance of the straight instrument being stopped at the depth of three inches and a half, and not appearing to be embraced by a tight canal, contradicted this idea. By perseverence, however, a curved instrument directed upwards, was passed into the bladder, and the finger in the rectum did not discover any intervening substance, but the mere walls of the gut and bladder—a thickness of four lines at most. There was no trace of scrotum, testicle, or of spermatic cord.

The mammæ were not at all developed. The young person declared that the cylindrical organ, or imperfect penis, was sometimes enlarged and erected, and the erections had occasionally been followed by a discharge of a whitish, thick, viscid fluid. The rami of the pubes and the tubera ischii were more approximated than usual; the hips projected; the buttocks flattened, and the thighs not rounded, and verging inwards as in females. The mons veneris, the linea alba, the chest, chin, and cheeks, and the extremities were covered with hair, and the general configuration of the body was decidedly masculine.

The medical men, after a most careful examination, concluded that the sex of the person had been mistaken, and the civil tribunal, therefore, ordered that the registry should be altered, and the surname changed to that of a male.—*Revue Medicale.*

25. *Probable Case of Extra-Uterine Pregnancy.*—Madame E. on the 26th Feb. 1817, was suddenly much terrified, *while receiving the embraces of her husband*, by the fall of a stone which had been thrown against the window of her bed-room. She had no family hitherto, though she had been married for 12 years. The catamenia had regularly appeared till after this date, when the other symptoms of pregnancy presented themselves. On the 13th April, she had a violent attack of bearing down, and of excruciating pain in the left side of the hypogastrium, which very closely simulated the threatening of an abortion. The symptoms gradually passed by, but again returned in two months. On the 6th Dec. all the signs of child-birth came on; but the physician could not, by examining, reach the os uteri with his finger. After a few hours of suffering, all pains left her, and from this moment the motions of the child, which had hitherto been very obvious, altogether ceased. For three months after this date, she had repeated attacks of inflammation in the abdomen, when, very unexpectedly, the catamenia re-appeared, and health was quickly re-established. The belly, however, remained very large. After two years of widowhood she again married, and enjoyed good health till April, 1824, when she died from an attack of peritonitis, followed by diarrhœa. On opening the abdomen, the fœtor was insupportable, and M. Beclard, therefore, contented himself by removing the tumour along with the uterus, to which it adhered, the fœtus was of the female sex and of the full size; it was enclosed in a bag, situated to the left of the womb, and was in a great measure coverted into a fatty matter, like adipocire.—*Archives Generales.*

26. *Torsion of the Arteries after Surgical Operations.*—Case I. A young man's leg was amputated by the circular incision. The anterior and posterior tibial, and the peroneal arteries, were twisted in the simple manner recommended by M. Amussat, without any previous laceration of the internal and middle tunics. The time occupied was much shorter than required for applying three ligatures; one of the tibial veins, which was bleeding profusely, was likewise twisted. There was an oozing, more serous than bloody, from the wound for several days. The wound did not heal favourably, as the patient's health was not good; in six weeks, however, the stump was quite cicatrized.

Case II. A woman, aged 48, had her thigh amputated; the femoral artery, well detached from the accompanying nerve, was twisted—only one other vessel required torsion. On the fourth day the bandages were removed; they were dry and very little stained; the wound was quite healed in four weeks.

This patient having subsequently died of phthysis, an opportunity was afforded of examining the vessels; the femoral artery was closed exteriorly by condensed cellular membrane, and plugged inwardly by a fibrinous white clot. Its walls and calibre were quite normal.

Case III. A man, aged 52, was admitted into the hospital with an enormous diffused, aneurismatic swelling of the lower third of the thigh: amputation was performed at the upper third. The femoral artery was attempted to be twisted, but it gave way at the third turn of the band, leaving a gaping orifice; a ligature was, therefore, applied —the muscular branches were, however, secured by torsion. For several days after the operation, there was an inconsiderable serous oozing from the wound, which, however, was completely cicatrized in about six weeks.

Case IV. A man, aged 24, had cancer of the penis, which required removal by the knife; five arteries were twisted. It was found much more difficult to detach these vessels from their connexions than in the previous operations. The wound healed within a month.

In addition to the above cases, several others are alluded to, of amputations, in which complete union of the wound took place in 6, 8, 12, 15, and 18 days.—*Journ. Hebdom.*

27. *Permanent Contraction of the Fingers cured by Dividing the Palmar Aponeurosis.*—Dupuytren has now performed this operation with complete success in upwards of twelve cases: and very lately he had a most satisfactory opportunity of confirming his opinion of the cause of this malady by dissection. An old woman died in the hospital; and it was found that three of the fingers were contracted. The skin of the hand and of the fore-arm having been very carefully dissected off, the aponeurosis was observed very distinctly to be drawn together. Wishing to shew that the distortion of the fingers was not at all dependent on any lesion of the tendons, Dupuytren divided them across, one by one; but yet the fingers could not be straightened; a direction was now carefully introduced beneath the palmar fascia, and this divided; and immediately the fingers could be bent back to their natural position—*Ib.*

28. *Capsicum in Hemorrhage.—To the Editor of the Boston Medical and Surgical Journal.*—Sir,—If you could induce Professor Tully or any one of your correspondents who is familiar with the article, to furnish you with a dissertation upon *capsicum*, you might probably be of more service to the profession than by giving to the public the same quantity of matter upon almost any other subject. It has long been pretty extensively and very advantageously employed as a gargle, in angina maligna, and other affections of the throat. It is also considerably used in some complaints of the stomach, and in the latter stages of most typhoid diseases. I have however, considered it as more valuable in *passive hemorrhage* than perhaps any single article of the materia medica. In this case, it may be employed in doses of three to five grains in pill, and may be repeated every ten or fifteen minutes, to the third or fourth time, in almost any instance, of a sudden profuse loss of blood, whether it is from the lungs, uterus, nose, stomach, or rectum. It generally answers when given alone, but its effect is usually more certain when each dose is combined with one or two grains of sugar of lead, and half a grain or a grain of opium.

Notwithstanding capsicum is so very important in hemorrhage, I do not know that its stiptic property is mentioned in the common treatises upon materia medica; and I suspect that the number of practitioners who employ it in this way is very limited.

Should this hint serve to call forth some abler pen to furnish a dissertation upon the subject, my end will be answered. SENEX.

29. *The Ornithoryncus Paradoxus.*—The following interesting fact in natural history was communicated by Dr. Weatherhead to the committee of science of the Zoological Society at their last meeting.

For the last five-and-twenty years naturalists in Europe have been striving to obtain the carcase of the impregnated female *Ornithorhyncus Paradoxus,* for the purpose of ascertaining its mode of gestation, but without success; for it is by dissection alone that the hitherto doubtful and disputed point concerning the anomalous and paradoxical manner of bringing forth and rearing its young can be satisfactorily demonstrated. This long-sought for desideratum is at length attained. Through the kindness of his friend, Lieut. the honorable Lauderdale Maule, of the 39th regiment, Dr. Weatherhead has had the bodies of several *ornithorhynchi* transmitted to him from New Holland, in one of which the ova were preserved; establishing, along with the other curious circumstances ascertained, the extraordinary fact, that this animal, which combines the bird and quadruped together in its outward form, lays eggs, and hatches them like the one, and rears and suckles them like the other.—*Proc. Zool. Soc.*

30. *Curious Anomaly in the Distribution of the Bloodvessels.*—The infant was born with imperforate anus, and lived for seven days. Dissection shewed that the rectum terminated in a cul de sac, about an inch above the os coccygis, which was but imperfectly developed. From the arch of the aorta, was given off, first, a trunk common to the two carotids; next the left subclavian; and lastly the right subclavian, arising from the extremity of the arch, and then crossing to the right side between the œsophagus and the vertebræ; the abdominal aorta having supplied the cœliac, superior Mesenteric, right renal, (the left was wanting, and likewise the kidney of this side) and spermatic vessels, divided into two branches on the second lumbar vertebræ. One of these, by far the largest, and appearing to be the continuation of the trunk, gave rise to the inferior mesenteric, and then crossed to the posterior surface of the bladder, along the median line of the umbilicus, where it divided into two. The other branch, situated behind the former, first followed the course of the sacrum, then was reflected upwards to the right sacro-iliac synchondrosis, having first supplied branches to the left lower extremity, and left side of the pelvis. The continuation of this vessel formed the right femoral. The system of the sympathetic nerves appeared to follow the irregularities of the arterial distribution—there being only one sacral ganglion, and no coccygeal.

M. Jodin, who has reported this case, is of opinion that the imperfect development of the rectum was dependent on and caused by the imperfect development of the pelvic blood-vessels, and very properly directs the attention of medical men to examine whether the connexion always exists.—*Archives Generales.*

31. *Dissection of a person who died from Inanition.*—A prisoner at Toulouse died after having refused to take food for 63 days.

Universal emaciation; weight of the body about 58 pounds. Brain of a paler color than natural; no water in the ventricles; cortical substance of healthy consistence; medullary substance of the cerebrum, cerebellum, and the medulla oblongata, considerably firmer and denser than is usually observed. Heart of the ordinary size, but very pale in color; soft and easily torn; lungs nearly normal; œsophagus contracted; stomach of its ordinary size, and containing an ounce or two of greenish fluid; mucous membrane adhering strongly; the thickness of the walls of the stomach and intestines sensibly diminished; valvulæ conniventes very distinct; hardened fæcal matter in the right portion of the colon; the rest of the intestines empty. The omentum reduced to a mere serous web, which was traversed by blood-vessels. Mesentery containing no fat. Liver of a healthy color and size; its substance denser than usual. Gall-bladder distended with a black, thick, bile, which was like a strong solution of extract of liquorice. Spleen exceedingly small, almost round, with a diameter of about two inches; texture very dense and resisting pressure. Kidneys reduced in size, and of very firm consistence. Muscular system almost "annihilated," so shrivelled were the fleshy "ropes of life." Though no trace of fat was to be observed, the marrow was not deficient in the long bones.—*ib.*

32. *Tumour—the remains of an old Hernial Sac in the Sheath of the Spermatic Cord.*—A man, aged 29, had a tumour in the spermatic cord, situated between the inguinal ring and the testicle, of the size of a walnut. It has been there for fifteen years, and given little annoyance till three months ago, when it began to increase in size and to become more painful. When carefully examined, it conveys to the finger the sensation of an enclosed fluid; the ring, cord, and testicle are not involved with it. The patient states, that at first the swelling might be pushed up to, but never within, the original canal. It was considered to be either an encysted tumor of the cord, or the remains of an omental hernia, or a fleshy or scirrhous tumor. M. Roux extirpated it. On puncturing it, a quantity of pale yellow serum flowed out. Death, from fever, took place a fortnight after the operation, and on dissecting the inguinal canal, the omentum was found adhering all round to its circumference. M. Roux gave it as his opinion that the tumor which was removed, had been an old hernial sac, the open neck of which had been in course of time obliterated; and stated what tended to confirm this was, that during the operation it was found to be very much more coherent at its upper than at its lower part.—*Jour. Hebd.*

33. *Retention of Urine caused by a Foreign Body in the Rectum.*—A man, aged 67, had suffered for several months from tenesmus and strangury, the urine dribbling away very slowly, and the desire to void it being very frequent. Many surgeons had attempted, but in vain, to sound him, with a view of curing a supposed stricture. On examining the rectum with the finger, a hard substance was discovered, pressing by one of its ends on the urethra; by the other on the coccyx, when removed it was found to be the bone of a partridge, which, from the patient's account, must have lodged there for two months at least. He was immediately restored to health.—*Archives Generales.*

34. *Arresting Hemorrhage.*—Dr. Arentz, of Norway, recommends nitric acid as the most powerful means of arresting hemorrhage. In bleeding from a vessel too deeply seated to be easily accessible, or in false aneurism, he pours eight or ten drops of nitric acid into the wound.—*Casper Critisches Reporter.*

35. *To protect Iron and Steel from Rust.*—Heat the object until it burns the hand; after which, rub it with very white wax. Heat it a second time in order to melt off the wax, and then rub it briskly with a piece of cloth or leather to impart to it brilliancy. This operation renders the metal proof against rust from exposure to the atmosphere.—*Recueil Ind.*

